# High-Altitude Structural Landmark Perception and Crosstalk Filtering Using Multiple LiDARs for Autonomous Driving Environments

**DOI:** 10.3390/s26175462

**Published:** 2026-08-28

**Authors:** Dokon Kim

**Affiliations:** Department of Smart Mobility, Pyeongtaek University, Pyeongtaek 17869, Republic of Korea; dokon@ptu.ac.kr

**Keywords:** multi-LiDAR, LiDAR perception, autonomous driving, LiDAR clustering

## Abstract

High-level autonomous driving requires an exceptionally accurate and robust perception of surrounding road environments to support reliable vehicle localization. While utilizing multiple LiDAR sensors expands the field of view and provides high-density point clouds, it introduces severe challenges, such as multi-LiDAR mutual interference (crosstalk) and significant computational overhead. This paper proposes a multi-stage front-end perception pipeline designed for robust high-altitude structural landmark extraction and crosstalk suppression to provide clean geometric reference points for downstream positioning systems. The proposed framework first employs a Binary Bayes Filter combined with a mesh-graph representation to segment stable overhead road traffic signs while minimizing environmental noise. To ensure real-time operation, a hybrid cascade consisting of 2D-grid spatial projection and voxelized 3D Density-Based Spatial Clustering of Applications with Noise (DBSCAN) is introduced, drastically reducing point cloud density bottlenecks. Finally, a cluster-refinement stage is applied to filter out residual false positives induced by crosstalk. The system was validated using real-world driving data collected over an urban sequence (1.88 km) and a highway sequence (37 km). Experimental results demonstrate that the pipeline achieves high landmark tracking success rates of 79.9% and 98.5%, respectively, while suppressing crosstalk-induced false alarms down to 12.3%. Operating entirely on CPU threads with an average latency of 24.5 ms per frame, the framework satisfies real-time execution bounds for standard 10 Hz LiDAR setups, establishing a high-fidelity front-end capable of preventing tracking drift in autonomous vehicle localization.

## 1. Introduction

Precise feature extraction and localization in dense urban and highway environments remain fundamental challenges in autonomous driving [1]. While traditional filtering frameworks—such as standard Kalman filters, Probability Hypothesis Density (PHD) filters, and conventional Occupancy Grid Maps (OGMs)—have achieved success in static object tracking, they struggle with high-density noise and dynamic occlusions. In multi-LiDAR setups, inter-sensor interference (crosstalk) introduces severe transient artifacts [2], while dynamic traffic frequently obscures ground-level landmarks.

To resolve these challenges, this paper presents a novel processing framework specifically designed for robust high-altitude landmark extraction. By targeting overhead road traffic signs (e.g., gantries and elevated cantilever structures), our approach leverages occlusion-free spatial cues to ensure reliable localization.

The primary contributions of this work are three-fold:

1. Cascaded Low-Latency Architecture: We propose a computationally efficient 2D-Grid to 3D-Voxelized DBSCAN cascaded architecture that drastically reduces searching space complexity from $O\left(N^{2}\right)$ to $O\left(N\log N\right)$, enabling robust high-altitude landmark extraction in under 30 ms strictly on CPU hardware.
2. Joint Crosstalk Suppression Mechanism: We develop a dual-stage artifact removal framework that synergistically pairs a temporal Binary Bayes Filter with high-altitude mesh-graph topological continuity analysis to eliminate multi-sensor optical mutual interference without dropping valid target points.
3. Real-World Validation: We validate the end-to-end framework across 38.8 km of complex urban and highway environments, demonstrating superior accuracy and real-time suitability compared to state-of-the-art baselines.

### Terminology Taxonomy and System Scope

To guarantee technical precision and prevent conceptual ambiguity across the manuscript, we establish a rigorous terminology taxonomy that governs the functional boundaries of our pipeline:

1. Landmark Perception/Extraction: Refers strictly to the front-end processing stages—including 2D-Grid spatial pruning, 3D Voxelized DBSCAN, and dual Bayes-mesh filtering—that detect, isolate, and crop high-altitude structural point clouds from raw sensor streams.
2. Spatial Clustering: Refers exclusively to the point-grouping algorithms (DBSCAN and mesh-graph topological grouping) that form coherent 3D candidate clusters from unorganized point clouds.
3. Landmark Data Association/Tracking: Denotes the frame-to-frame matching mechanism that maintains temporal identity and tracks extracted landmark candidates across consecutive sweeps.
4. Downstream Localization: This strictly refers to external vehicle-level state estimation backends (ego-pose SLAM/EKF) that receive our extracted landmark centroids as geometric reference inputs.

In accordance with this taxonomy, terms such as ‘recognition’, ‘clustering’, ‘tracking’, and ‘localization’ have been systematically audited and standardized throughout all sections. Furthermore, the entire text has undergone comprehensive language polishing to eliminate redundant phrasing, improve transition flow, and maintain consistent academic prose.

## 2. Methods

In autonomous vehicles, tracking techniques are applied to detect and track objects to ensure stable object estimation from various noises in the surrounding environment. Among various methods for representing dynamic objects in tracking techniques, a common approach utilizes multiple Kalman filters [3]. Kalman filters, which are based on single-object tracking, assume that only one measurement exists per track. However, in many situations, simultaneous tracking of multiple objects is required, and inaccurate or incorrect measurements can make it difficult to determine which measurement to assign to which track. Therefore, several approaches that enable multi-object tracking using multiple Kalman filters or particle filters have been proposed over the past several decades, with data association being a representative approach. The Probability Hypothesis Density (PHD) filter models multiple objects and measurements as a set of objects and a set of observations based on a random finite set. Unlike existing data association-based tracking methods, it estimates the state by considering objects as a single set rather than estimating each object independently [4]. Grid-based area map recognition techniques have been used to recognize the surrounding environment of autonomous vehicles. Area maps can represent surrounding objects in a grid format through probabilistic sensor modeling of recognized object information or sensor measurements. Area map recognition techniques have been proposed to recognize and track objects in the surrounding environment using various environmental sensors, such as LiDAR, radar, and cameras [5,6,7,8,9,10]. Additionally, sensor fusion research is being conducted to utilize the different characteristics of environmental recognition sensors to improve the recognition performance of a single sensor [11,12]. In local map environment recognition, the surrounding environment is represented using a static occupancy grid map (SOGM), which stores the presence or absence of static objects. However, the static grid map method has limitations in that it can only represent the location information of static objects on the grid map. This can lead to errors in recognition due to low occupancy probability in grids containing moving objects. Recently, research is being conducted on a technique that overcomes the limitations of the existing static grid map technique by using a dynamic occupancy grid map (DOGM), which can represent not only static but also dynamic obstacles on the grid map [13,14]. Dynamic grid mapping techniques have the disadvantage of requiring significant computational and memory resources as the dimensionality of the state being estimated increases. To overcome this, research on dynamic grid mapping using the Sequential Monte Carlo (SMC) method, which utilizes multiple GPU cores simultaneously to perform computations in parallel, is actively underway [15,16,17].

For object recognition, such as buildings and signs, high-quality point cluster information for high-altitude objects can be obtained through three stages: LiDAR data preprocessing, object clustering, and cluster refinement. First, in the LiDAR data preprocessing stage, points suspected of being noise are removed from the input point data based on their relationship to surrounding points centered around the point’s 3D coordinates. Among the points that are located at high locations for the first refined points, points that can form a mesh graph are segmented as high-altitude structure points. In this process, if the LiDAR sensor can receive signals by distinguishing between high-intensity threshold satisfied points and low-intensity threshold satisfied points, only the high-threshold points are used to construct a mesh graph. This process allows for obtaining point data that is robust to noise. Next, points that are determined to be the same object are clustered based on the attribute information of the segmented points to create clusters. One or more of the methods described below can be used for cluster creation. Finally, the clusters processed in the previous step are refined into cluster units. This process removes clusters that are misaligned due to noise generated by crosstalk effects inherent in the characteristics of LiDAR, as well as clusters that are noisy around high-altitude objects [18]. This study applied multiple clustering methods and various techniques that can be used in the refinement process to obtain high-quality object information, and these techniques are discussed in the remainder of this paper.

### 2.1. Point-by-Point Segmentation

**Figure 1:**
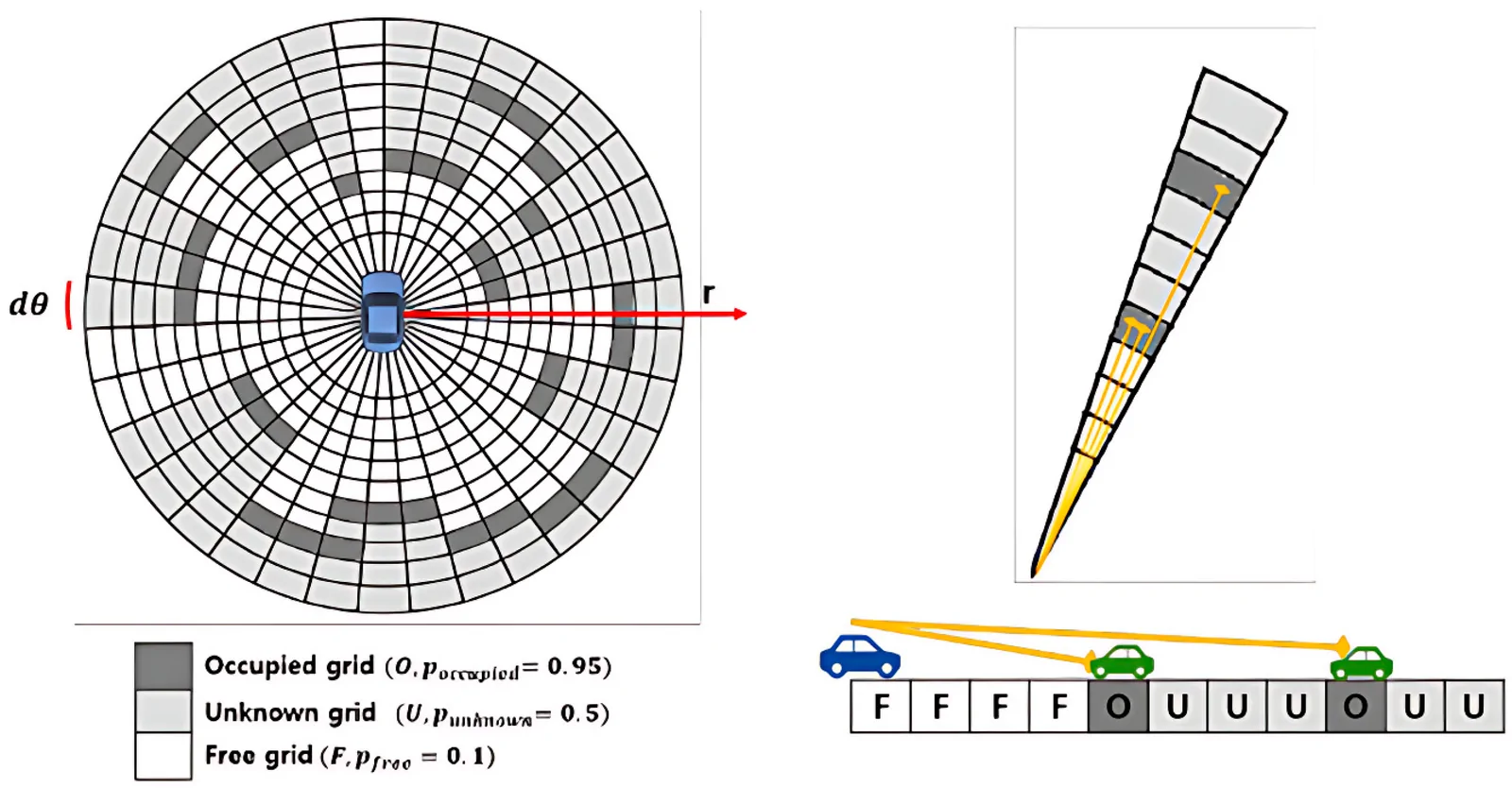
LiDAR sensor model.

LiDAR sensors are Time-of-Flight (TOF) sensors that measure distance to an object by measuring the time it takes for a measuring beam to bounce back after striking the object’s outline. Based on these sensor characteristics, measurement models typically include four types of error: measurement error, error due to dynamic objects, error due to failure to measure, and random error [19]. LiDAR probability models include forward and inverse sensor models, with the inverse sensor model typically used. In this study, the inverse sensor model of LiDAR was used, and the grid map constructed in polar coordinates was converted to a Cartesian coordinate system. Furthermore, due to the characteristics of the LiDAR mounting location, an inverse sensor model, as shown in Figure 1, was implemented to ensure that the object behind the nearest object is measured and its characteristics are reflected [20,21]. When changing from a polar coordinate system to an orthogonal coordinate system, the grid close to the vehicle contains multiple polar coordinate grids. One method for determining which polar coordinate grids to include in a single grid is to calculate the occupied area of the grid and assign a probability value to the grid by reflecting a weight based on the area. Furthermore, a sampling method can be used to model the probability of multiple polar coordinate grids in a nearby grid.

The number of samples (ns) per orthogonal coordinate grid can be obtained using the following Equation (1) [22]. In the equation, ρ is the distance from the origin O to the center coordinate (x, y) of the grid with size dx. dρ and dθ are the distance and angular resolution of the polar coordinate grid.

To determine the expected number of polar samples $n_{s_{x,y}}$ falling into a single Cartesian grid cell at position x,y, we calculate the ratio between the Cartesian cell area $A_{\mathrm{cartesian}}$ and the polar beam sector area $A_{\mathrm{polar}}$. Assuming a uniform square grid with resolution dx, the cell area is defined as $A_{\mathrm{cartesian}}=dx^{2}$. In polar coordinates, the differential spatial area at radial range $\rho =\sqrt{x^{2}+y^{2}}$ with radial resolution dρ and angular resolution dθ is represented as $A_{\mathrm{polar}}=\rho \cdot d\rho \cdot d\theta$. Consequently, the sample density factor $n_{s_{x,y}}$ is derived as

(1) $$n_{s_{x,y}}=\frac{A_{\mathrm{cartesian}}}{A_{\mathrm{polar}}}=\frac{dx^{2}}{\rho \cdot d\rho \cdot d\theta }$$

**Figure 2:**
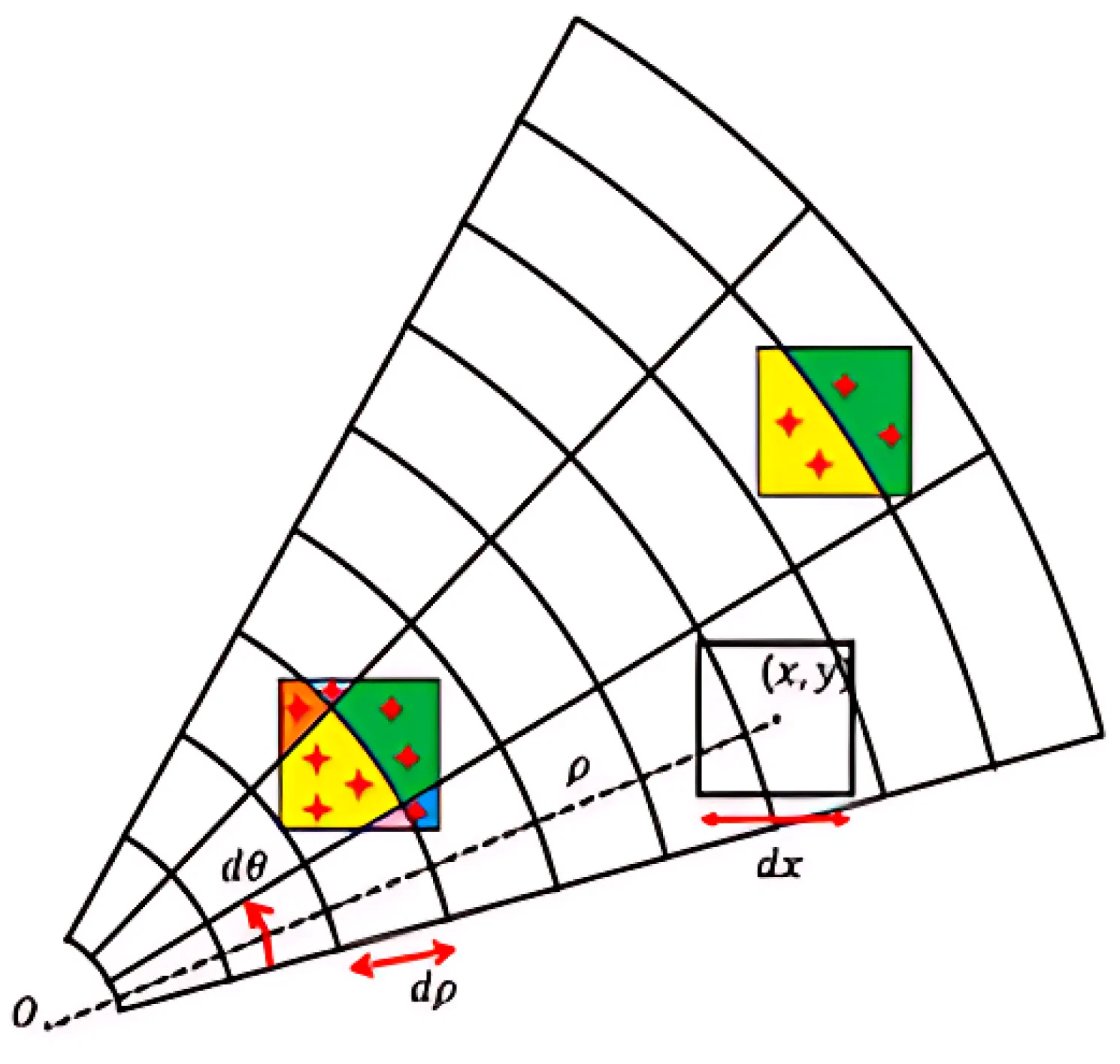
Polar to rectangular coordinate conversion.

As illustrated in Figure 2, this geometric relationship accounts for the spatial decay of polar measurement density as the range ρ increases from the LiDAR origin.

The probability of updating the grid map uses the Binary Bayes Filter (BBF). BBF is a method of representing the occupancy status of a grid with two states: occupied and free. A static grid map uses the occupancy probability of the grid in the previous step as the current predicted occupancy probability. The basic principle of BBF is that the state of each grid is assumed to be independent of the states of other grids, so BBF is calculated individually for each grid. As in Equation (2), the occupancy probability of the k + 1th grid, po,k+1 (ok+1), is calculated by calculating the posterior k occupancy probability po,k (ok) at time k and the measurement probability pzk+1 (ok+1|zk+1) expressed by the inverse sensor model to update the grid map.

(2) $$p_{o,k+1}(o_{k+1})=\frac{p_{\mathrm{zk}+1}(o_{k+1}|z_{k+1})\cdot p_{o,k}(o_{k})}{p_{\mathrm{zk}+1}\left(o_{k+1}|z_{k+1}\right)\cdot p_{o,k}\left(o_{k}\right)+p_{\mathrm{zk}+1}({\bar{o}}_{k+1}|z_{k+1})\cdot p_{o,k}({\bar{o}}_{k})}$$

**Figure 3:**
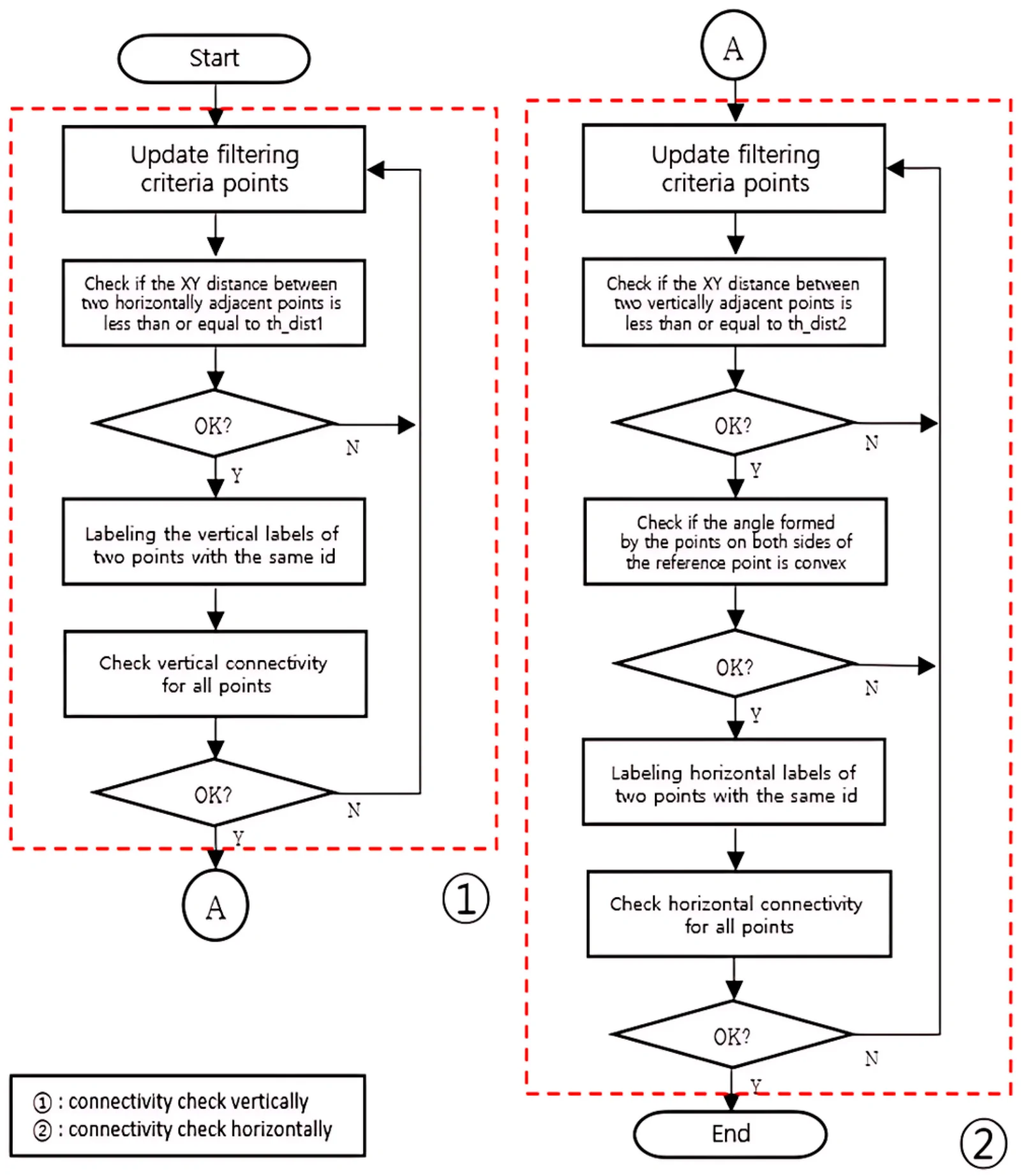
Flowchart for vertically and horizontally checking point connectivity for mesh graph construction.

**Figure 4:**
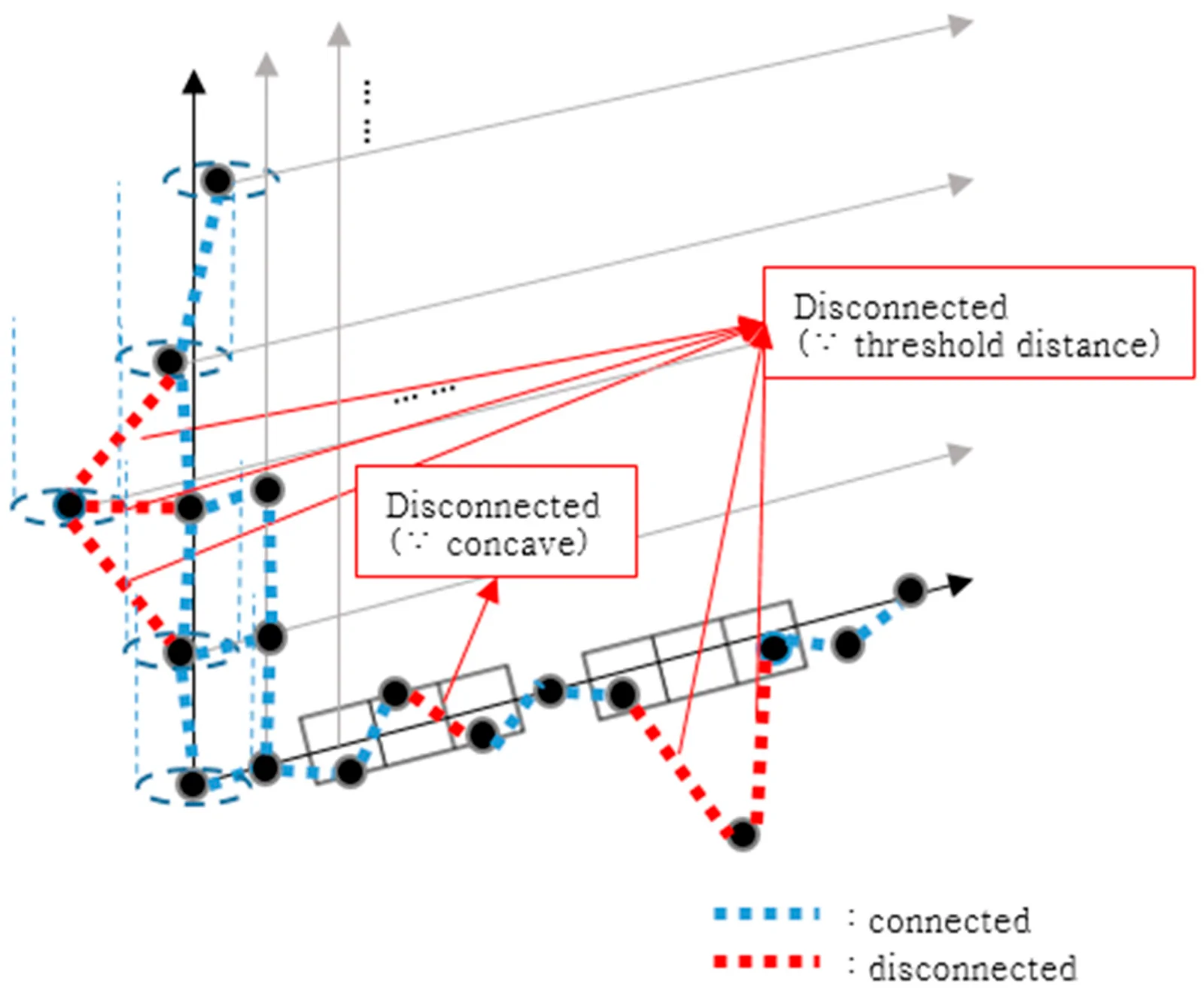
Example of mesh graph configuration using points.

The idea of a point-wise clustering method based on a mesh graph structure was utilized to detect points generated by overhead road traffic signs such as buildings [23,24,25]. The detailed procedure for vertically and horizontally checking point connectivity for mesh graph construction follows the flowchart illustrated in Figure 3. It is characterized by creating a mesh graph by connecting consecutive LiDAR points based on the firing order and layer structure of the LiDAR sensor and forming a cluster based on this (Figure 4).

In addition, one type of LiDAR used in this study can simultaneously acquire SW for up to two points through processing at the level of a single firing laser, which can obtain signals for high- and low-threshold points for objects with relatively strong reflectivity, and signals for low-threshold points for objects with relatively weak reflectivity.

In the case of such LiDAR, by utilizing the above characteristics, more accurate high-altitude structure points can generally be obtained. Therefore, in this study, we adopted a method to configure it robustly against noise that may occur in the sensor by using the default point if the LiDAR sensor does not enable signal processing according to a threshold through the above method, and using only high-threshold points if possible. Based on the given point, we determined whether the graph of points was connected by checking whether the characteristics between adjacent horizontally or vertically adjacent LiDAR points were similar to the characteristics of points appearing in overhead road traffic signs. As features, we utilized Euclidean shape information, such as the distance between points and whether the angle formed by three consecutive points satisfies the convexity.

**Figure 5:**
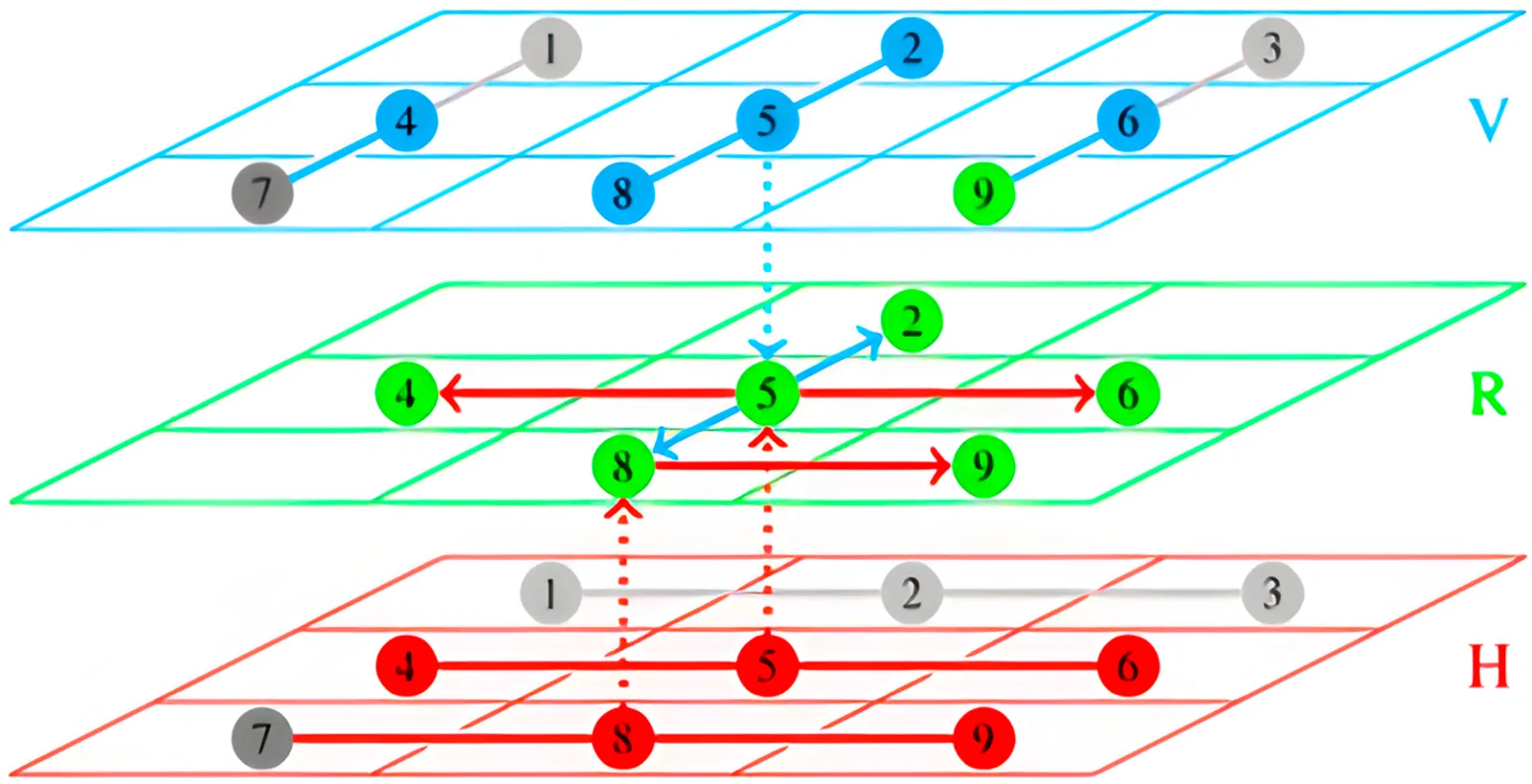
Vertically and horizontally labeling for mesh graph construction.

Afterwards, based on the mesh graph connected according to the characteristics of overhead road traffic signs, the connected points can be labeled with the same ID. At this time, the region-growing methodology is used for labeling, and the seed point is set to 4 m or higher, so that only structures above a certain height are labeled. The labeled points are those with the same horizontal labeling ID (H in Figure 5) or vertical labeling ID (V in Figure 5) as the point to be compared, and the same labeling ID is assigned to points connected with the same ID through region growing to expand the 3D area, and the labeled points can be segmented as high-altitude structure points.

### 2.2. Structural Clusters

Clustering refers to a method of grouping data with high similarity by considering the characteristics of the data, and placing data with different characteristics into different groups. DBSCAN is a method of clustering data based on the principle that data with similar characteristics are close together and distributed at a high density [26,27]. Because clustering is based on data density, it offers the advantage of clustering without a pre-specified number of objects, unlike the K-Means technique, which is a representative clustering method. However, clustering performance deteriorates when the density of data with similar characteristics is not uniform. DBSCAN classifies the data to be clustered into the following four categories using the distance condition εd and the minimum reference number nc as parameters.

- Neighboring data: Data whose distance from another data is closer than or equal to εd.
- Core data: Data with a number of neighboring data greater than or equal to nc.
- Peripheral data: Data that is not core data, but is neighboring to core data.
- Noise data: Data that has less than nc data within a distance εd, and all of those data are not core data.

**Figure 6:**
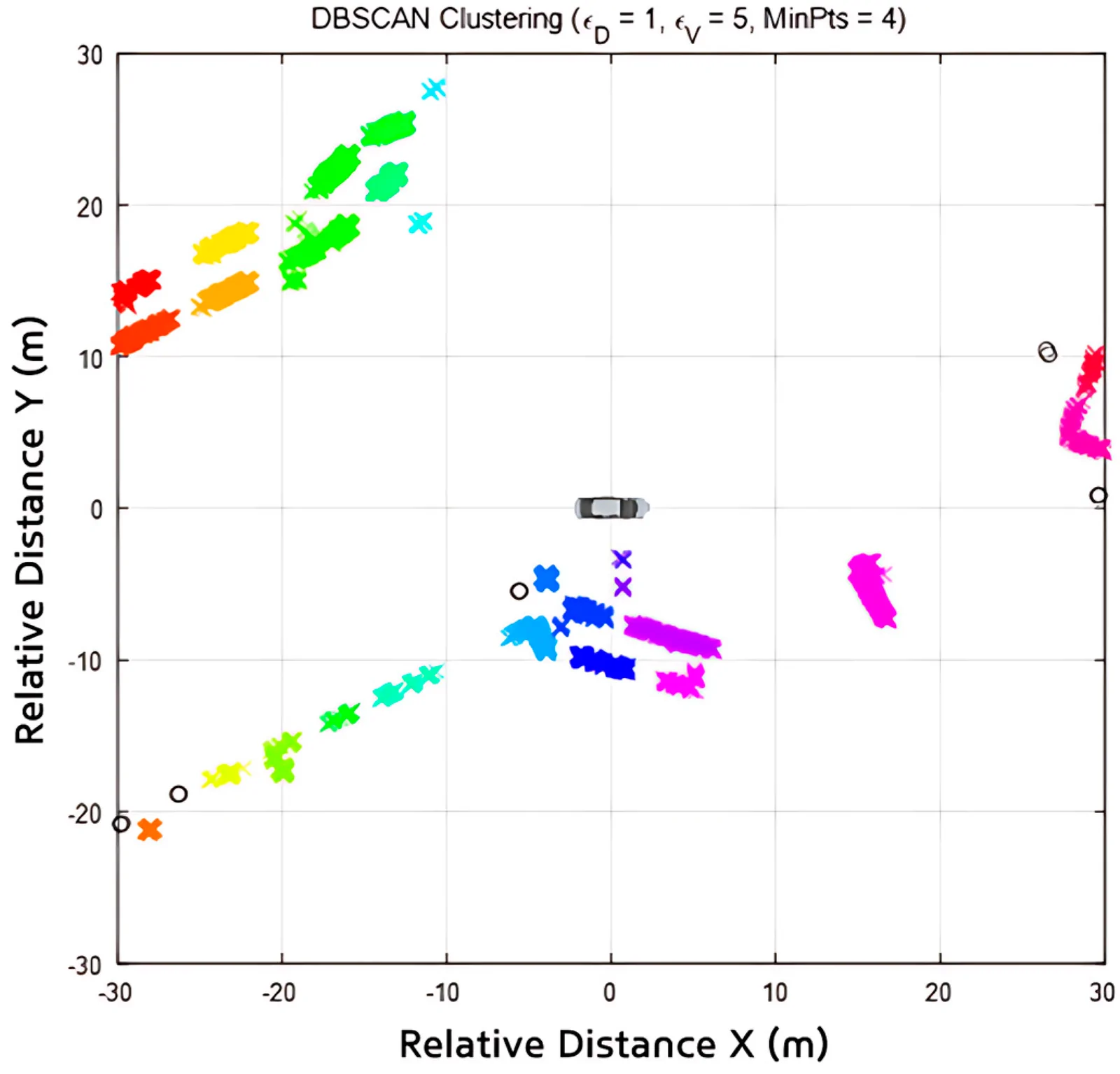
Clustering results obtained via the proposed Velocity−adaptive Grid DBSCAN.

DBSCAN classifies clusters and noise in two steps: exploring the surrounding space and counting the number of adjacent data. DBSCAN outputs individual clusters as labels y, and if the y values of two data points are the same, it means that the two data points are clustered in the same cluster. DBSCAN selects one data point x from the given data and then finds the neighboring data z within εd. If the number of neighboring data of x is greater than or equal to nc, x is classified as core data and a new cluster is formed. At this time, z, which is a neighboring data of x, is also assigned to the same cluster as x. Afterwards, the surrounding space is searched to find neighboring data of solution z, and if z is classified as core data, the neighboring data of z is also assigned to the same cluster as x. In this paper, we leverage the advantage of dynamic grid maps in estimating representative velocities as well as grid locations. We modify DBSCAN to extract neighboring data that satisfy both the εd condition, which represents distance differences, and the εv condition, which represents speed differences. This ensures that adjacent data with speed differences are clustered differently. Figure 6 shows the results of clustering the estimated grid state information (location, velocity) from the dynamic grid map using speed-based DBSCAN. In the clustering results, “o” indicates grids identified as noise, and “×” and color indicate individual clusters. It can be seen that static objects such as buildings and traffic signal controllers at intersections, as well as vehicles moving within the range of 10 to 20 m on the x-axis and −10 to 0 m on the y-axis, are individually clustered.

One or more LiDAR data were used simultaneously for high−altitude structure recognition depending on the number of LiDARs, installation location, and application system, and the appropriate method for each case was applied to reduce execution time while improving accuracy.

#### 2.2.1. Hardware Platform and Spatial Sensor Configuration

**Figure 7:**
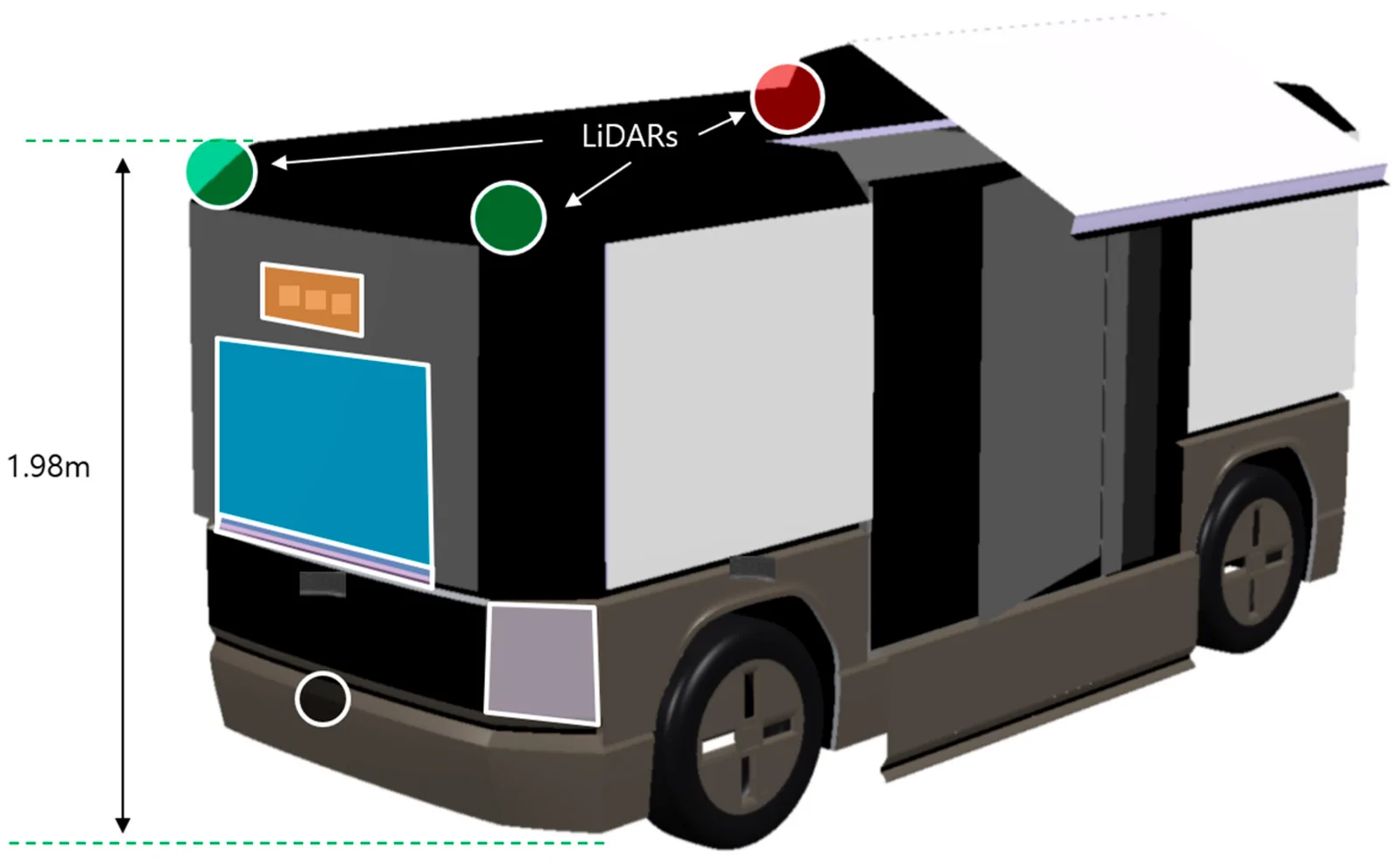
Geometric schematic and multi-sensor spatial configuration of the experimental platform.

To evaluate the proposed high-altitude landmark extraction framework under realistic driving conditions, an experimental vehicle platform was constructed. Due to the vehicle provider’s strict security and confidentiality policies, actual photographs of the vehicle exterior cannot be disclosed. Alternatively, the detailed physical configuration, sensor locations, and scanning geometry of the hardware platform are mapped out via the precise geometric schematic in Figure 7.

The primary 3D LiDAR sensor (Velodyne HDL-64E) was rigidly fixed to a customized roof rack at an exact mounting height of 1.98 m above ground level, aligned precisely with the vehicle’s longitudinal centerline. To expand the environmental scanning envelope and provide comprehensive structural coverage, two secondary 3D LiDAR sensors were deployed symmetrically on the front corners of the roof rack. The comprehensive hardware capabilities of these primary and secondary sensors are summarized in Table 1.

Ensuring the spatial synchronization of this multi-sensor array requires a precise understanding of their extrinsic geometric relationships. Instead of relying on manual adjustments, the rigid extrinsic transformations of the secondary sensors were calibrated relative to the coordinate origin of the primary central LiDAR. Specifically, the secondary front-left sensor was positioned at a relative baseline distance of Δx = 0.45 m, Δy = 0.60 m, and Δz = −0.05 m away from the primary origin, with an intentional orientation tilt configured at a pitch of 2.5° and a yaw of 15.0°. Symmetrically, the secondary front-right sensor was offset by Δx = −0.45 m, Δy = 0.60 m, and Δz = −0.05 m, featuring a pitch of 2.5° and a yaw of −15.0°. This specific geometric arrangement intentionally tilts the secondary sensors to maximize the capture density of peripheral overhead road traffic signs. However, this configuration introduces a significant spatial overlap between the fields of view (FOVs) of the adjacent sensors in the near-to-mid range. While beneficial for dense mapping, this overlapping FOV zone serves as the primary geometric trigger for the inter-sensor crosstalk, signal interference, and subsequent point cloud artifacts that are systematically analyzed and corrected in Section 3.1.

#### 2.2.2. Single LiDAR-Based Structural Mesh-Graph Clustering

**Figure 8:**
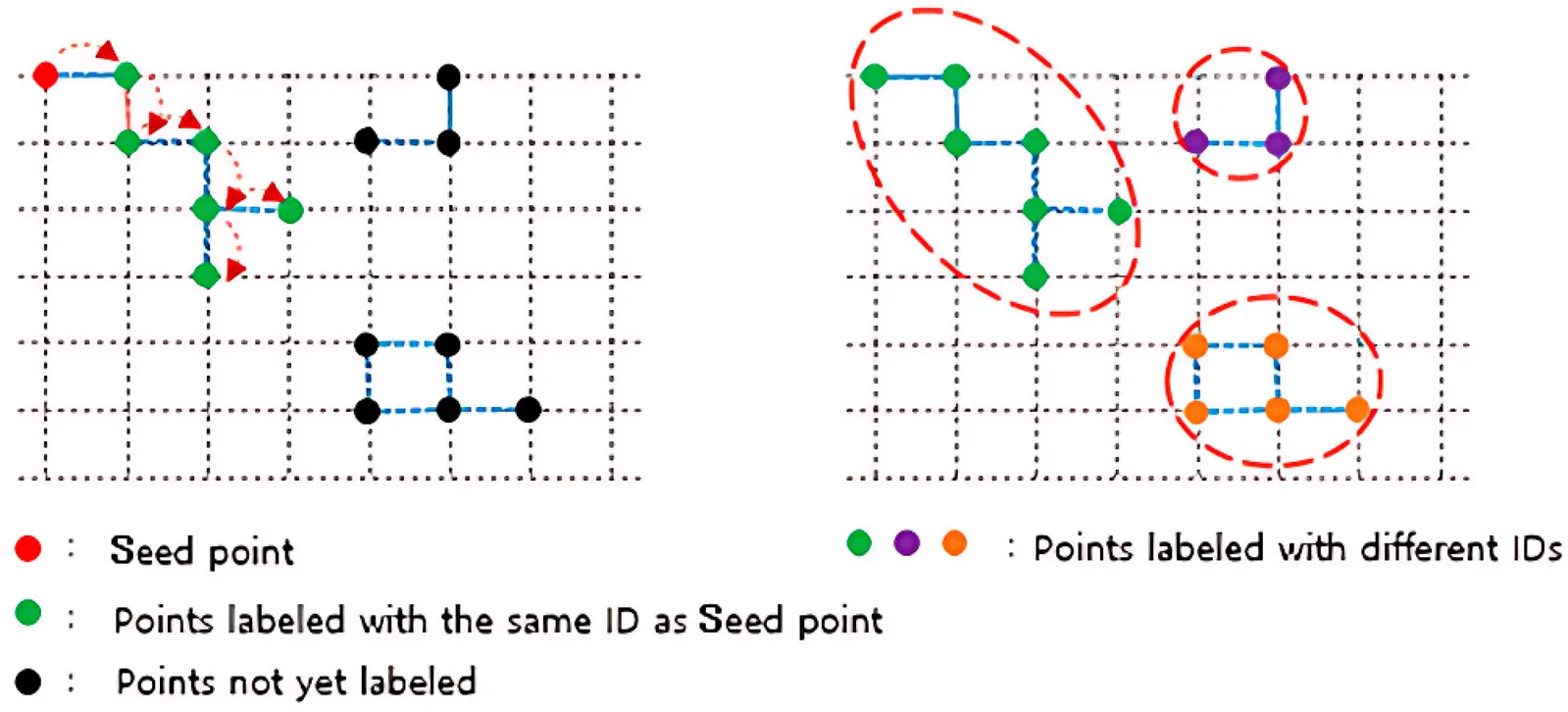
Points processed as clusters. The list of points belonging to each cluster is processed to update cluster attribute information (cluster height, length, height, etc.).

For vehicle configurations leveraging a single primary LiDAR sensor, the framework directly computes cluster attributes by evaluating the spatial connectivity of the constructed mesh graph. The mechanism of this point-level labeling and region-growing expansion is illustrated in Figure 8 (Points processed as clusters). As shown, a seed point is dynamically initialized at an altitude threshold of 4 m or higher to ensure that only structural elements elevated above the driving corridor are targeted. From this seed point, a region-growing algorithm recursively traverses adjacent nodes, assigning a uniform tracking ID to all topologically connected points that satisfy the geometric convexity and distance criteria.

**Figure 9:**
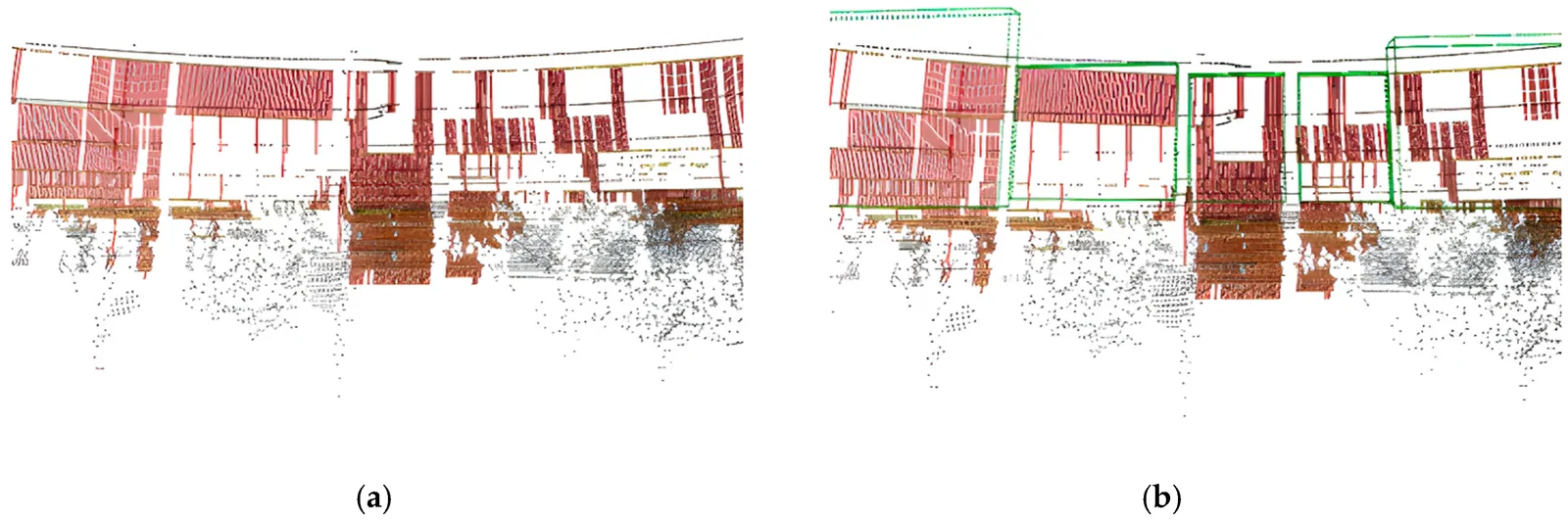
Clustering results for a single LiDAR dataset. (**a**) A mesh graph constructed based on point data. (**b**) A cluster labeled and clustered using region-growing.

The qualitative performance of this single-sensor processing phase is demonstrated in Figure 9. Specifically, Figure 9a visualizes the initial structural mesh graph constructed directly from the raw point sequences based on the sensor’s firing order and layer structure. Following the application of the region-growing clustering algorithm, Figure 9b highlights the final segmented clusters, in which high-altitude road structures are successfully isolated and bounded, proving the efficacy of the mesh-graph representation in preserving structural consistency under localized point sparsity.

**Figure 10:**
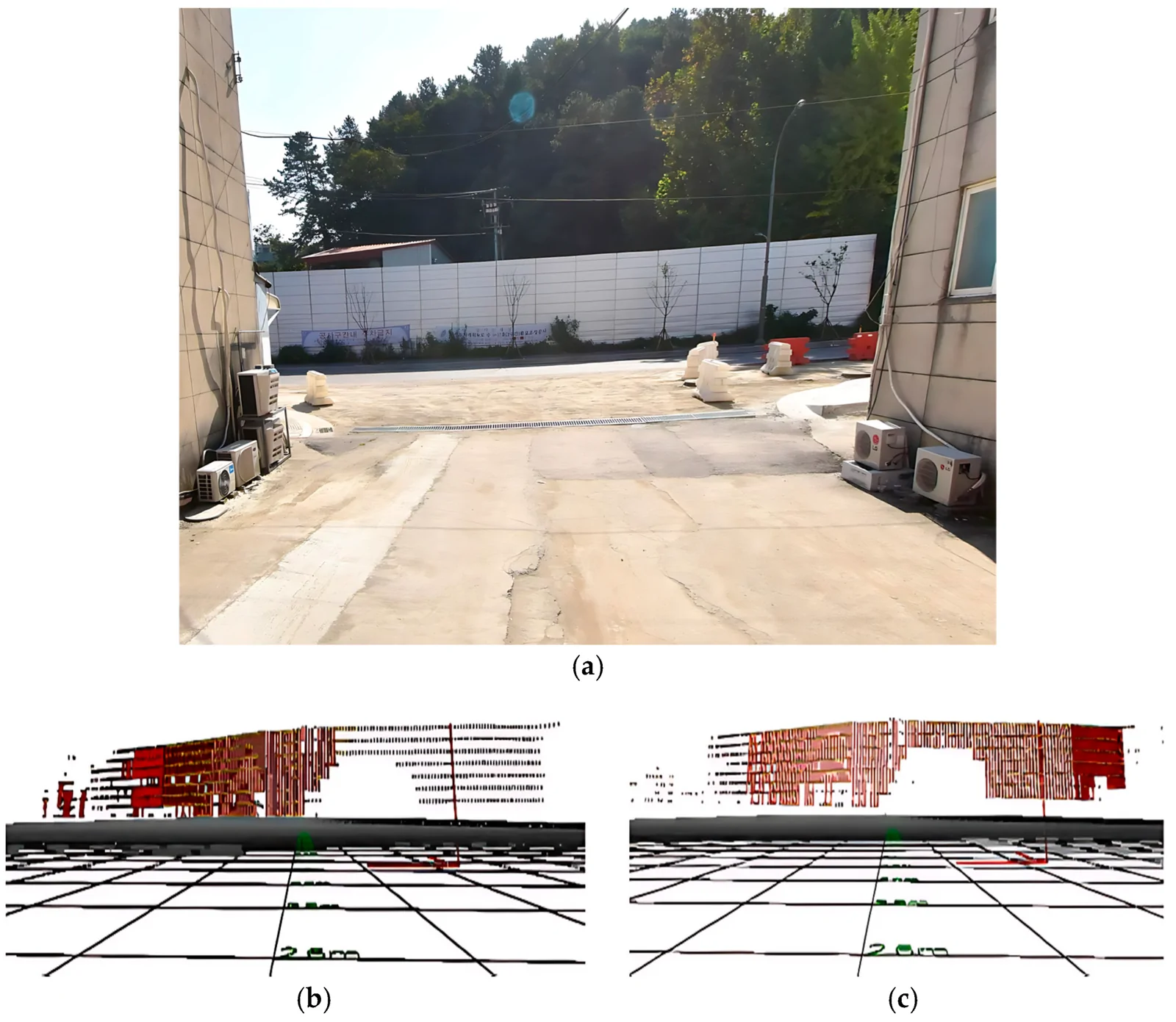
Camera data for the front barrier and mesh graphs for each sensor. (**a**) Camera data. (**b**) Mesh graph for the left sensor, no mesh graph formed to the right of the vehicle center. (**c**) Mesh graph for the right sensor, no mesh graph formed to the left of the vehicle center.

Furthermore, the inherent constraints of independent single-sensor processing within a multi-LiDAR array are highlighted in Figure 10. While the camera image in Figure 10a establishes the ground truth of a forward-looking spatial barrier, Figure 10b,c expose how the left and right LiDAR sensors generate highly disjointed, unilateral mesh graphs due to physical occlusions and asymmetric fields of view. This spatial fragmentation underscores the critical limitation of relying solely on single-sensor mesh structures and motivates the multi-LiDAR fusion strategy detailed in the subsequent subsection.

#### 2.2.3. Grid-Based Spatial Projection and Hybrid DBSCAN Re-Clustering

**Figure 11:**
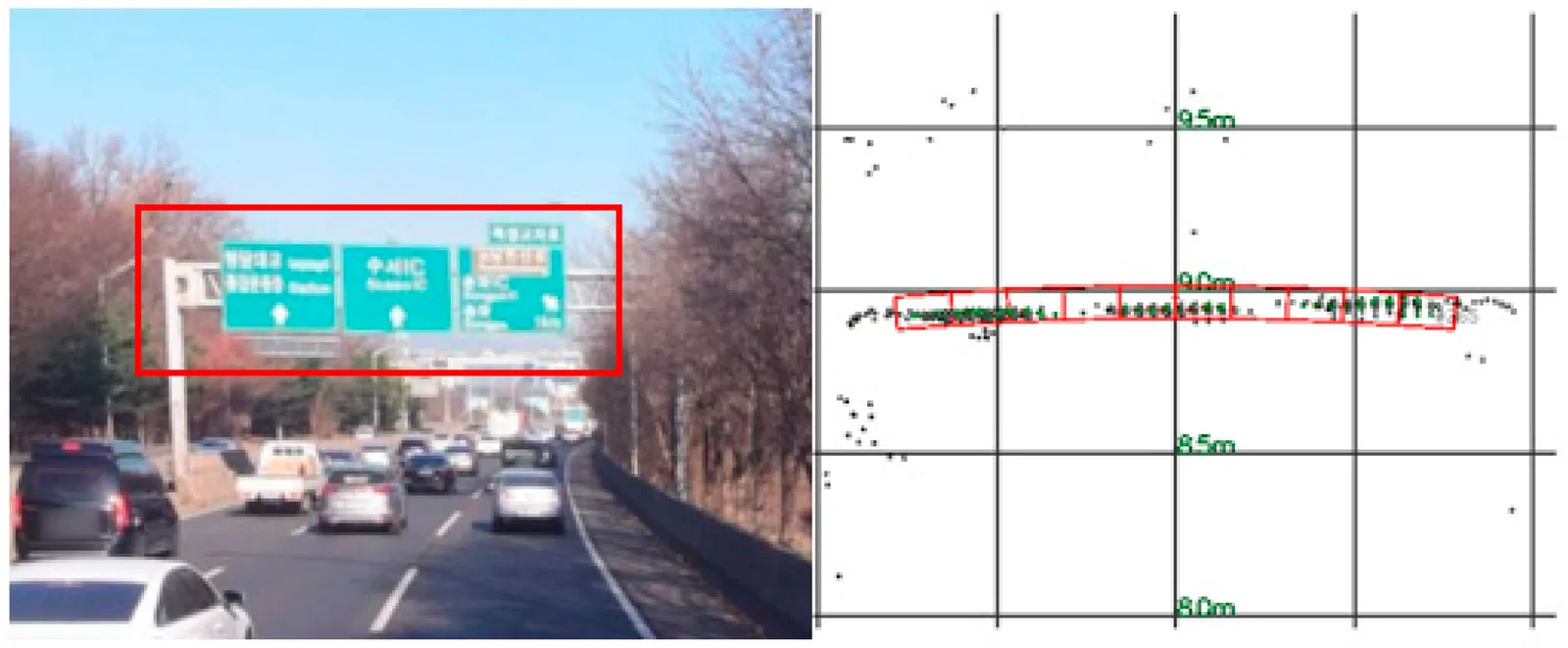
Example of applying a 2D grid to points segmented by high-altitude structure points.

To resolve the spatial fragmentation caused by multi-sensor occlusion, the framework projects the labeled high-altitude points from all individual sensors onto a unified, discrete topological space. Figure 11 illustrates an example of this spatial discretization, where the segmented high-altitude structure points are mapped onto a continuous 2D horizontal grid network to initiate a coarse-level region-growing expansion across overlapping fields of view.

**Figure 12:**
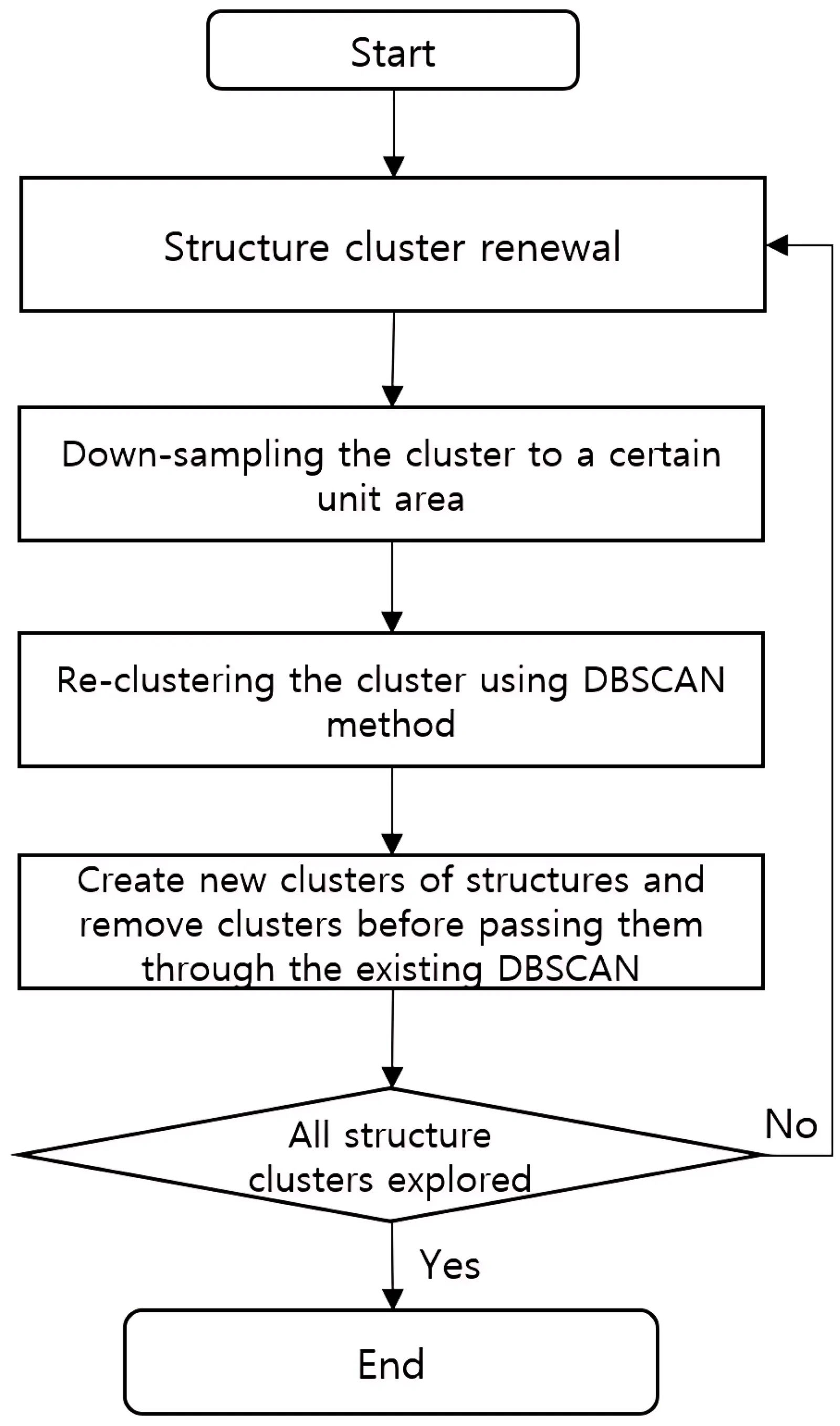
Flowchart of clustering Overhead Road Traffic Signs using two or more LiDARs.

The comprehensive, step-by-step execution of this multi-LiDAR re-clustering pipeline is systematically mapped out in the flowchart of Figure 12. To circumvent the high computational overhead typically associated with point-level density calculations, the pipeline introduces a hybrid cascade: points are first down-sampled into uniform unit voxels, and a secondary, fine-grained re-clustering is executed exclusively within these voxelized domains using the modified DBSCAN algorithm.

**Figure 13:**
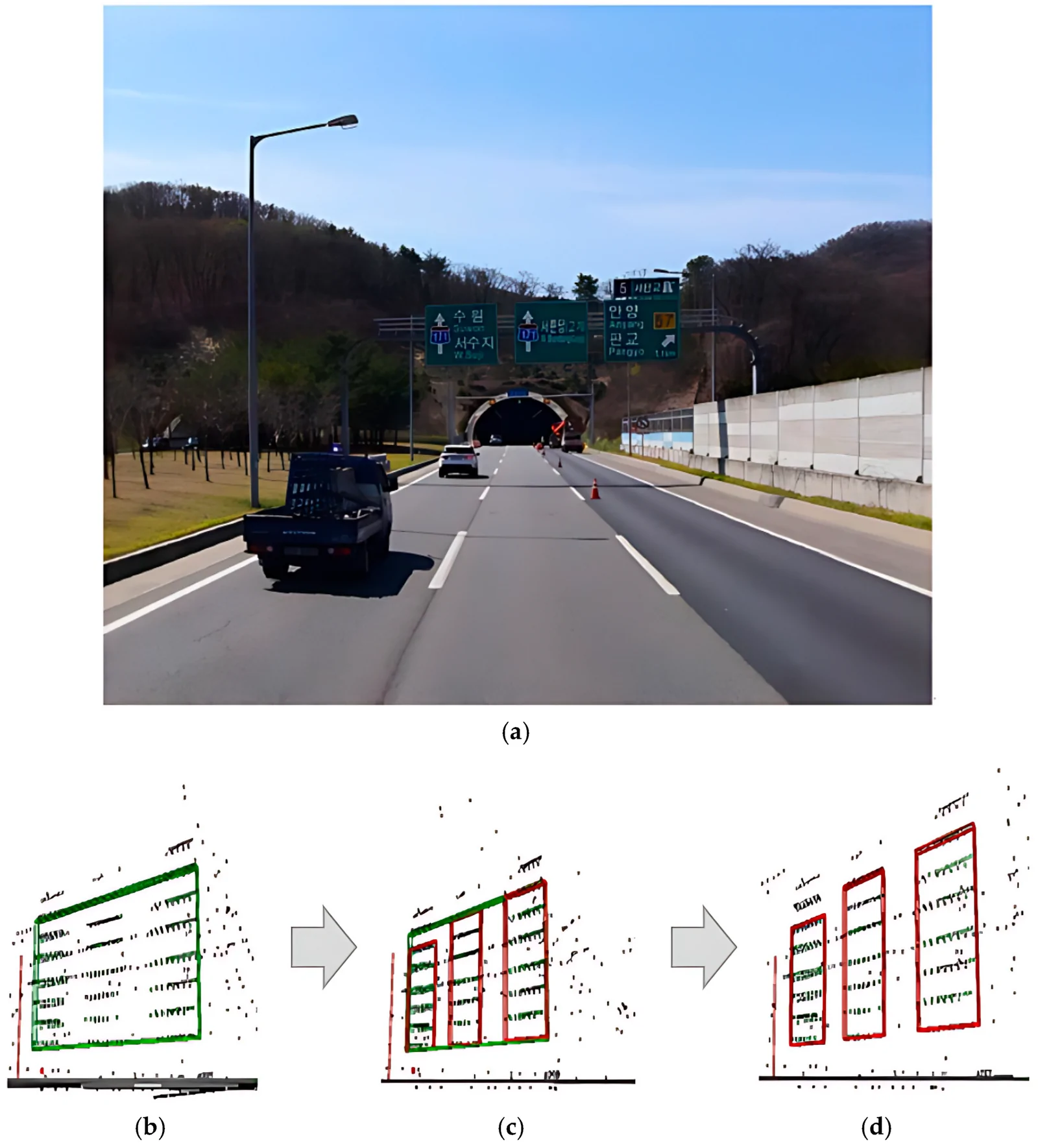
Example of camera and DBSCAN postprocessing for a forward-looking high-altitude structure. (**a**) Camera data. (**b**) Clusters generated by clustering using a 2D grid. (**c**) Re-clustering of the high-altitude structure clusters using DBSCAN. (**d**) Final high-altitude structure cluster output after removing the clusters generated by the 2D grid.

A qualitative breakdown of this cascaded processing sequence and its impact on landmark fidelity is demonstrated in Figure 13. Figure 13a provides the optical camera reference of a forward-looking highway signboard structure. The initial coarse clusters generated via the baseline 2D grid projection are visualized in Figure 13b, which exhibits severe geometric discretization errors and includes localized noise artifacts. As shown in Figure 13c, the application of our voxel-based DBSCAN re-clustering successfully partitions the dense structural cores at a high resolution. Finally, Figure 13d presents the optimized high-altitude structure cluster output after completely purging the grid-induced boundaries, ensuring both real-time execution and high geometric fidelity.

However, when a 2D grid is applied, the actual location of the point is not accurately reflected by the resolution of the grid, so when a cluster is created, it may appear as a single cluster with other nearby objects, or a false cluster may be created in which some noise is also clustered.

To overcome these limitations, DBSCAN was applied to clusters generated by grid-based clustering. DBSCAN is characterized by its robustness against noise and accurate results by grouping clusters with high density and close proximity at the point level.

However, DBSCAN performs clustering at the point level, which typically results in slower execution times compared to grid-based methods. To improve this, we performed DBSCAN using voxel-based quantization of the target points. This approach ensured real-time performance and improved the quality of the DBSCAN results [28]. The DBSCAN parameters (N, ε) used here were determined to achieve optimal performance based on actual driving data from the target route. To achieve this, data on areas where DBSCAN re-clustering could be effective was first acquired from the actual road data, and a 2D lookup table was then constructed based on this data.

In addition, the size of the unit voxel was also applied as the minimum area that satisfies the limited execution time among those that produce the desired high-altitude structure clustering performance based on actual data. In the vehicle configuration used in this study, two LiDARs mounted on the front side were used to recognize overhead road traffic signs ahead. The experimental data collection was conducted using a multi-sensor configuration consisting of three Velodyne HDL-64E LiDAR units. To eliminate temporal discrepancies and ensure accurate point cloud registration, all sensors were synchronized via the IEEE 1588 PTP (Precision Time Protocol) framework [29], achieving sub-microsecond timestamp alignment against a central grandmaster clock. Furthermore, spatial coordinate alignment between the sensors was established through a targetless extrinsic calibration routine, which precisely determines the relative transformation matrix by optimizing the registration of shared environmental geometric features.

#### 2.2.4. Topological Cluster Refinement and Crosstalk Suppression

**Figure 14:**
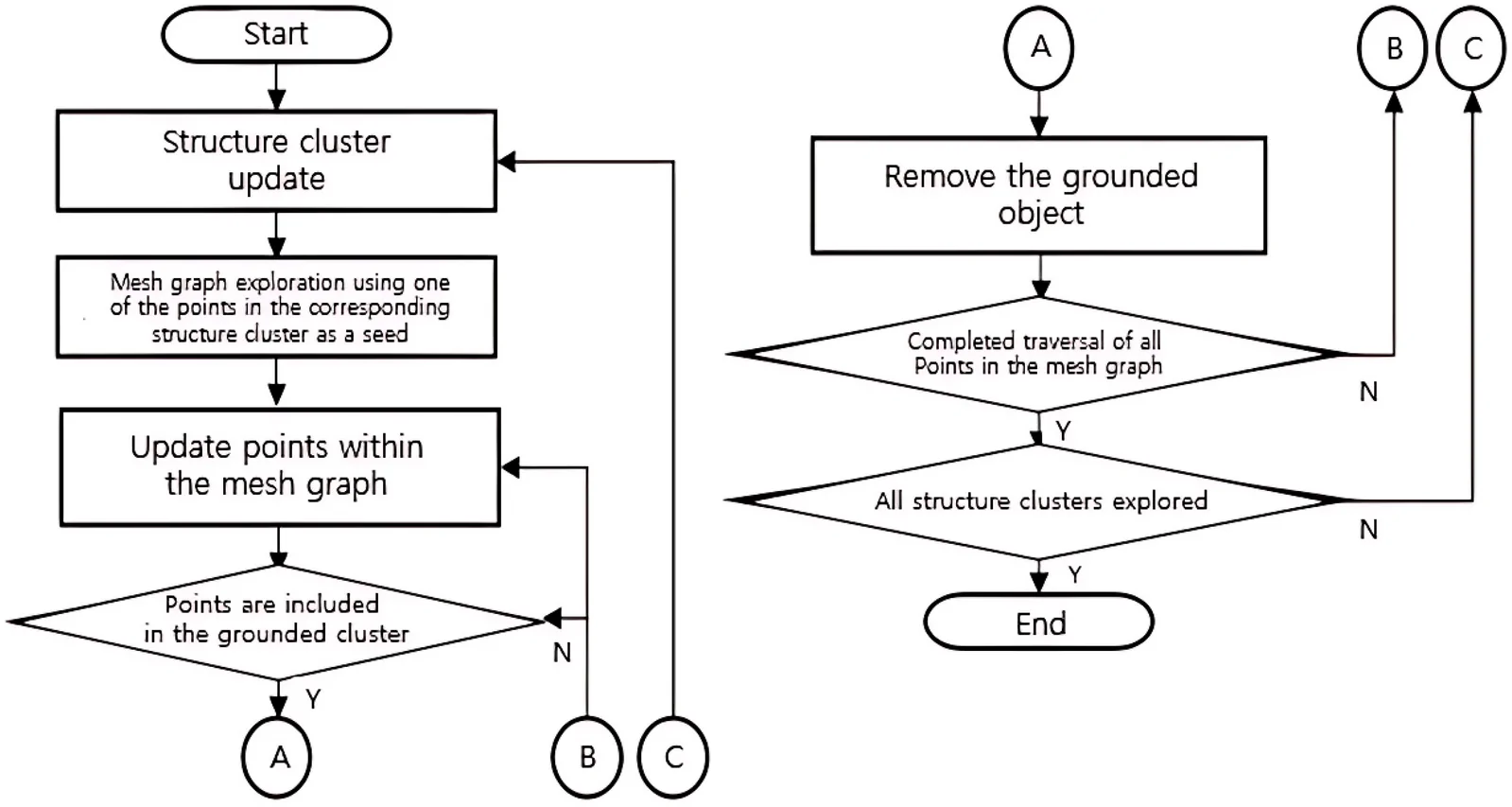
Cluster purification process flowchart.

The final stage of the perception pipeline focuses on mitigating mutual sensor interference and filtering out residual ghost clusters or distorted geometries induced by multi-sensor crosstalk. The systematic logic governing this purification stage is detailed in the flowchart presented in Figure 14, which evaluates the structural interplay between the primary mesh graph and the downstream clustered bounding volumes.

**Figure 15:**
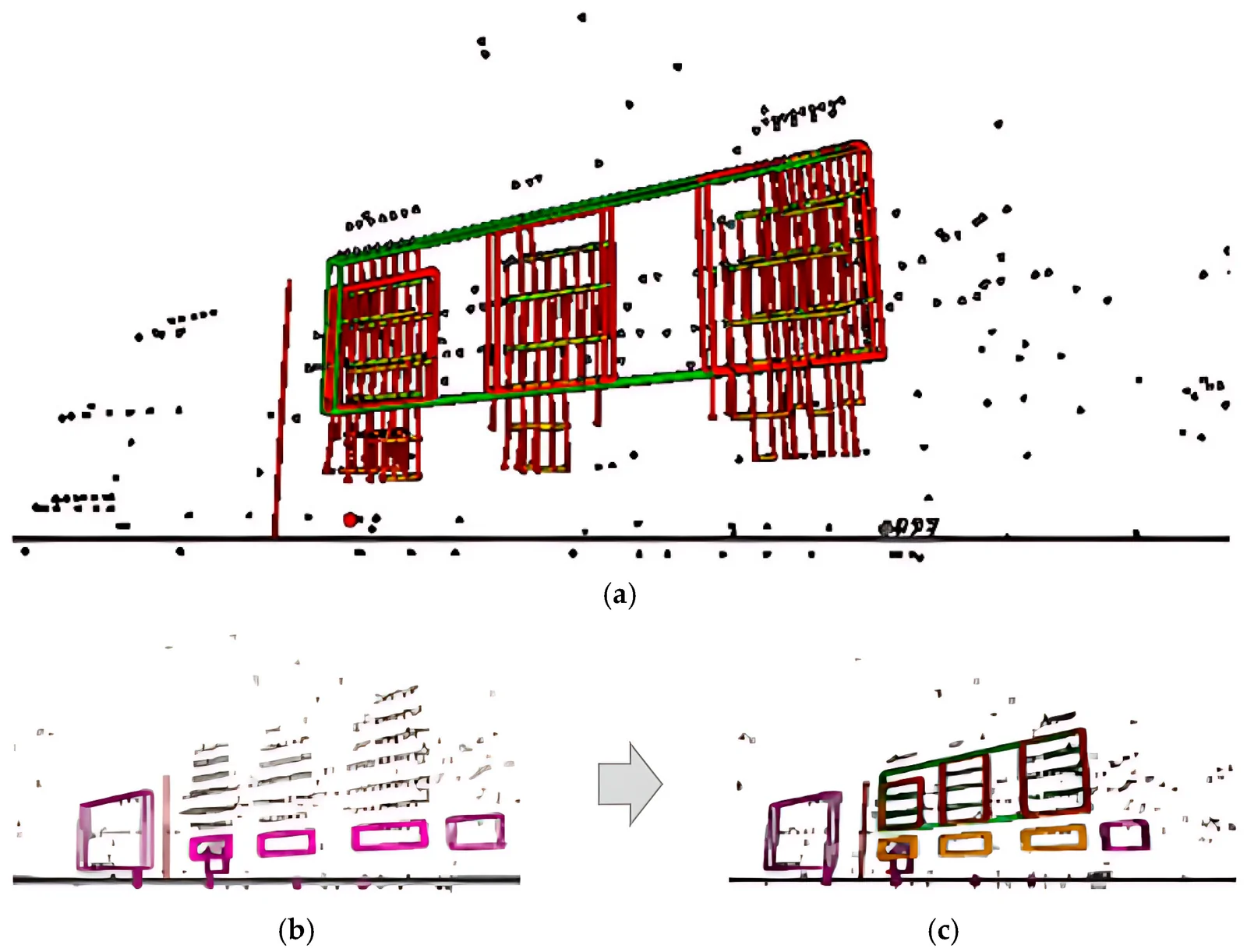
Example of cluster refinement. (**a**) High-altitude structure cluster and mesh graph. (**b**) Before cluster refinement, a grounded cluster is created below the sign due to crosstalk. (**c**) After cluster refinement, the grounded cluster (orange box), connected to the high-altitude structure cluster via the mesh graph, is removed.

A qualitative demonstration of these crosstalk suppression capabilities is provided in Figure 15. Figure 15a establishes the baseline high-altitude landmark cluster alongside its corresponding topological mesh graph. Figure 15b visualizes a prominent failure mode common in multi-LiDAR fleet operations prior to refinement, where un-synchronized stray pulses create a false, grounded ghost cluster directly beneath the target signboard. As demonstrated in Figure 15c, by leveraging the connectivity constraints of our proposed mesh-graph refinement, the framework successfully identifies and severs these false topological links. The erroneous grounded cluster (highlighted by the orange bounding box) is entirely eliminated, isolating the precise geometric centroid of the overhead signboard and confirming the pipeline’s robustness against severe environmental noise.

### 2.3. Methodological Novelty and Systematic Comparison

While individual operations such as grid partitioning, voxelized DBSCAN, and Bayes filtering are established techniques in point cloud processing, their conventional standalone applications fail to concurrently satisfy real-time CPU performance (<30 ms) and high-altitude feature preservation under severe multi-LiDAR crosstalk.

Standard 3D DBSCAN incurs prohibitive neighbor-search overheads ($O\left(N^{2}\right)$) when processing dense unorganized point clouds, making CPU-only real-time execution intractable. Conversely, 2D grid-based methods achieve high speed but discard critical elevation topography required for overhead structures. Our framework bridges this gap through a structural cascade: the 2D occupancy grid rapidly prunes ground and out-of-interest regions, passing only candidate spatial clusters to a localized 3D Voxelized DBSCAN.

Furthermore, optical crosstalk in multi-sensor setups manifests as transient, unorganized point clusters in overlapping field-of-views (FOVs). Standard spatial filtering techniques often misidentify these artifacts as valid obstacle boundaries or accidentally prune sparse overhead signs. To address this, our framework couples temporal probability updating with spatial topology. The temporal log-odds occupancy score $L_{t}\left(m_{i}\right)$ for cell $m_{i}$ given sensor measurements $z_{1:t}$ is updated as

(3) $$L_{t}\left(m_{i}\right)=L_{t-1}\left(m_{i}\right)+\log\left(\frac{P\left(m_{i}|z_{t}\right)}{1-P\left(m_{i}|z_{t}\right)}\right)-L_{0}\left(m_{i}\right)$$

Transient optical crosstalk fails to accumulate temporal confidence ($L_{t}\left(m_{i}\right)<{\mathrm{th}}_{\mathrm{occupancy}}$) over consecutive frames. For residual transient noise within single frames, the mesh-graph structure evaluates the local vertex degree distribution $K_{v}$. True structural landmarks exhibit coherent edge connections, whereas crosstalk ghosts lack topological continuity ($K_{v}<{\mathrm{th}}_{\mathrm{degree}}$) and are effectively pruned. Table 2 summarizes the structural comparison between existing paradigms and our proposed framework.

## 3. Methodology Validation

Positioning accuracy for autonomous driving demands centimeter-level precision. LiDAR is a sensor that emits highly linear infrared light and precisely extracts target location information based on the reflected light. Therefore, many positioning techniques utilizing LiDAR are being studied [30,31,32,33,34,35,36,37]. Particularly, advanced frameworks have recently integrated immediate structural meshing and representation graphs to guarantee both centimeter-level landmark tracking and high frame rates [38]. Various static road environment structures exist on roads, and utilizing these diverse road environment structures can yield robust positioning results. Signs, streetlights, curbs, and lane lines are static structures that can exist on roads. Their shape and reflectivity can be used to extract features for feature-based positioning. Various methods, such as edge extraction, continuity testing, clustering, and normal vectors, can be used to extract shape features from data measured by LiDAR [39]. Signs and lane markings are painted with highly reflective paint to improve visibility. The paint contains glass beads, so the light illuminating the signs and lane markings is retroreflected, resulting in relatively high reflectance [40].

**Figure 16:**
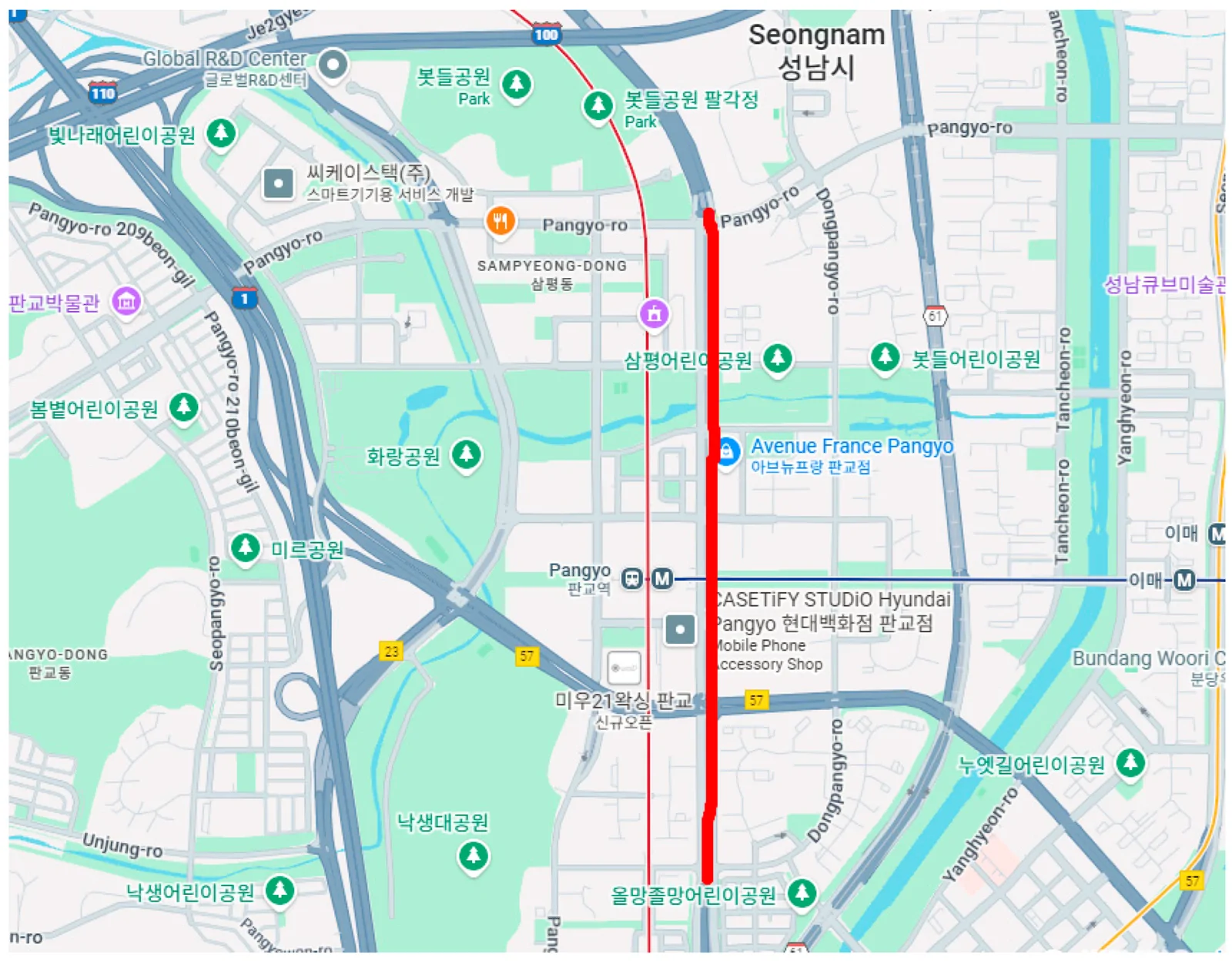
Some of the test sections.

To verify the feasibility of the previously applied methods, a portion of the test section was selected for evaluation using the under-development LiDAR for mapping against a high-precision map. For quantitative evaluation, each sensor and system under development for autonomous driving development was considered. The roof LiDAR was used for building recognition in urban areas, and the forward-facing LiDAR was used for landmark recognition among overhead road traffic signs. Furthermore, while verifying the recognition performance of high-altitude objects, cluster refinement was performed to address the impact of crosstalk effects. Based on experimental data, the impact of crosstalk effects was reduced. To verify the results of high-altitude building recognition, the vehicle drove along a designated route. The existing high-precision map for this route allowed for the verification of the clustering quality of overhead road traffic signs. This route is part of the test section shown in Figure 16. The total driving distance was 1.88 km, and the number of frames was 1818. To prevent numerical accumulation of duplicate data, the performance for high-altitude clustering was analyzed for 902 frames in which the vehicle speed was estimated to be 3 kph or higher.

**Figure 17:**
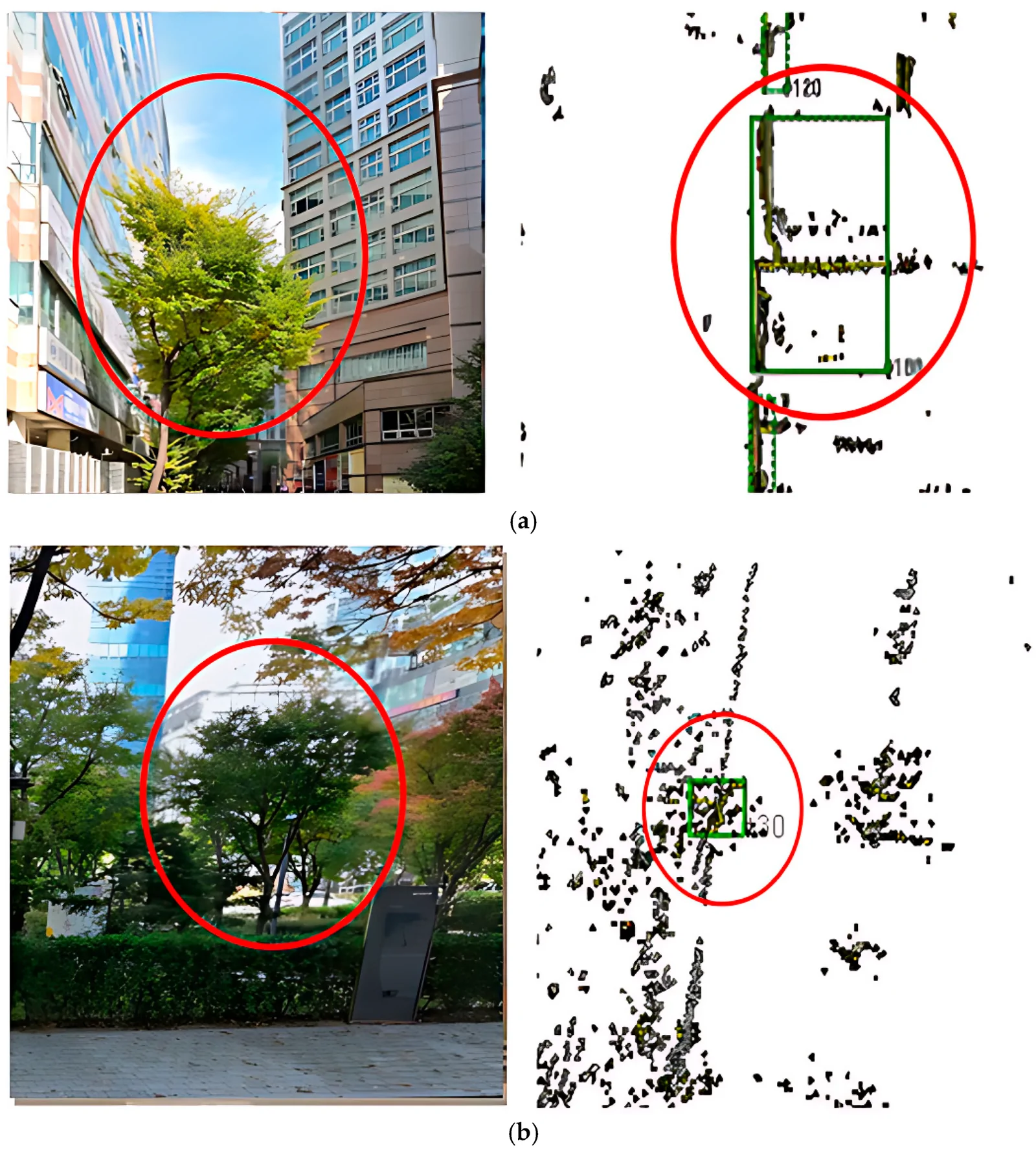
Description of a case where building recognition malfunctions in an urban area. (**a**) Two buildings appear as a single cluster of overhead road traffic signs due to the presence of a structure between them. (**b**) A cluster of overhead road traffic signs appear even though it is not the desired high-altitude structure.

Among these, 101 frames contained multiple buildings as a single high-altitude cluster, and 80 frames contained a cluster of overhead road traffic signs, even though these were not the intended overhead road traffic signs. This evaluation identified 181 frames (20.07% of the total valid frames) that exhibited environmental noise or potential artifacts capable of negatively impacting positioning accuracy if left unfiltered. Conversely, by successfully neutralizing these anomalies, the remaining 721 frames (approximately 79.93%) were processed with high geometric fidelity. Consequently, these robustly clustered frames (approx. 80%) are expected to stably contribute to the downstream positioning and localization systems without introducing tracking drift (examples of building recognition malfunctions are shown in Figure 17). Next, we examined the recognition results for overhead road traffic signs. The target road traffic sign recognition area was selected as a landmark located between 70 and 120 m from the vehicle’s path, a relatively easy-to-detect area.

**Figure 18:**
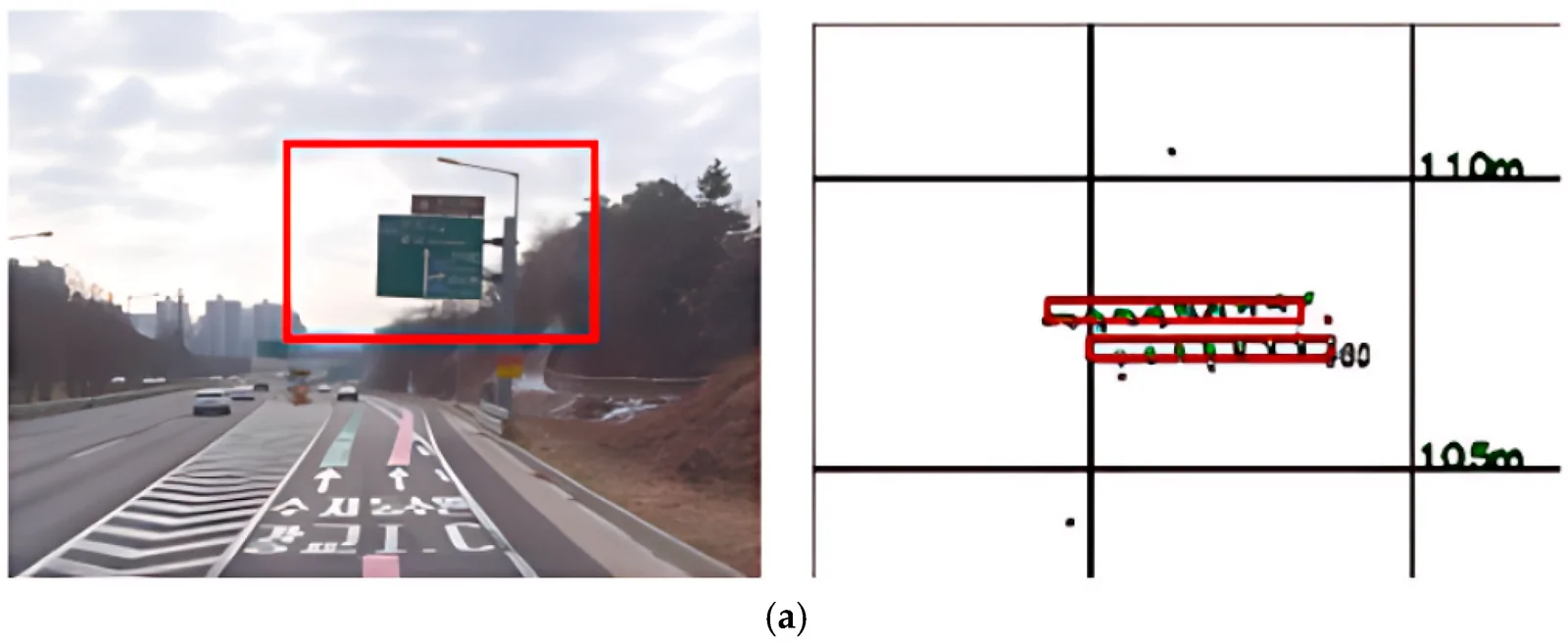

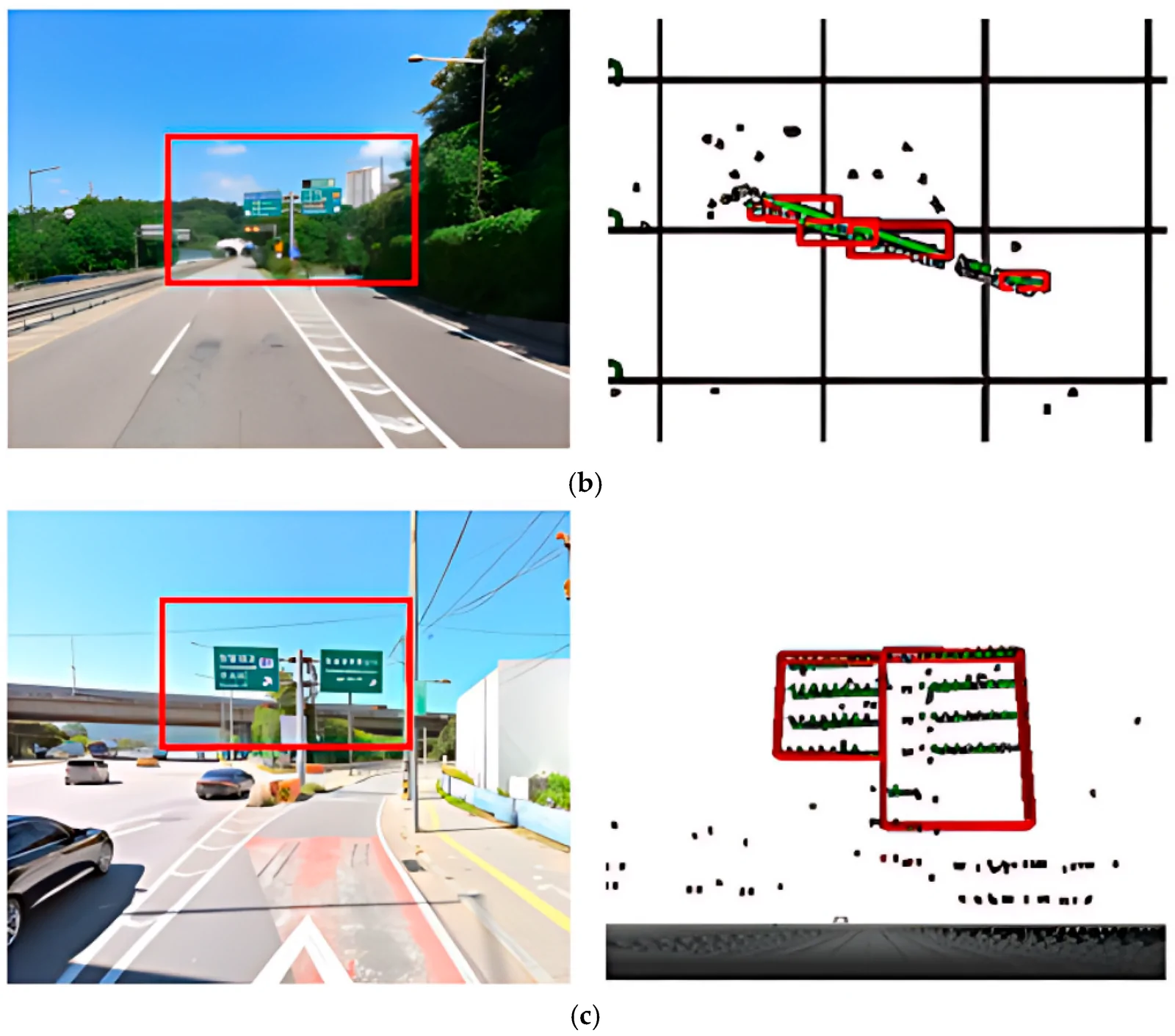
Description of a case of landmark recognition malfunction. (**a**) The left and right sensors are out of sync, causing each sensor’s points to form separate clusters. (**b**) The landmark is not perpendicular to the vehicle’s direction of travel, resulting in multiple clusters. (**c**) The structure to which the landmark is attached appears large, clustered with the landmark.

In the 37 km section consisting of 36,570 frames, target overhead road traffic signs were detected in a total of 2844 frames. Among these, clusters were extracted smaller than the original objects in 2802 frames. Additionally, multi-sensor optical mutual interference (crosstalk) caused spurious structural clusters in 32 frames, while non-perpendicular sensor angles in turning sections led to multiple clusters in 3 frames. Finally, large clusters integrated with background structures were observed in 7 frames. In addition, there were 62 frames in which a landmark existed at the corresponding location but was not detected because it was obscured by the object in front. Out of the 2844 evaluation frames, 2802 frames (98.52%) were classified as acceptable for geometric reference tracking. To evaluate the tracking performance under diverse driving environments, these 2844 frames were categorized into three distinct sub-scenarios based on the road characteristics: Urban Area (1000 frames), Expressway (1000 frames), and Complex Intersection (844 frames). Although these 2802 frames included instances where the extracted clusters were segmented slightly smaller than the physical boundaries of the original objects due to distance-dependent point sparsity, they still preserved the critical structural centroids and vertical alignment features. Because these primary geometric descriptors remained intact and stable enough to serve as reliable registration landmarks for the positioning system, they were included in the successful recognition margin, yielding a robust tracking rate of 98.5% (common landmark recognition malfunctions are illustrated in Figure 18).

**Figure 19:**
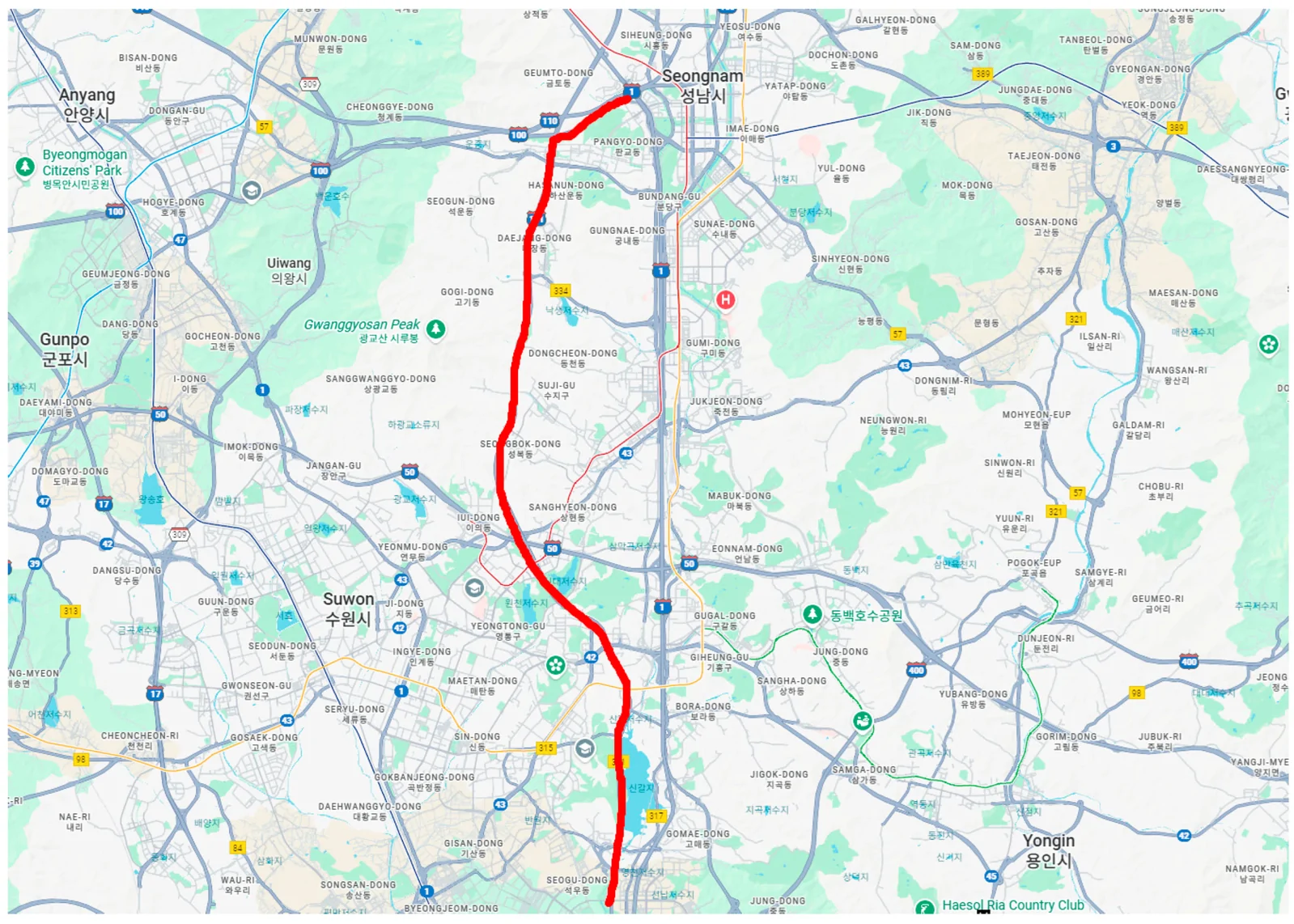
Cluster refinement test section for detecting clusters of overhead road traffic signs and minimizing the impact of crosstalk effects.

Finally, we verified the effectiveness of removing false objects generated by the crosstalk effect. To verify this, we acquired and verified data for the section of the Yongin expressway, highlighted in red in Figure 19.

**Figure 20:**
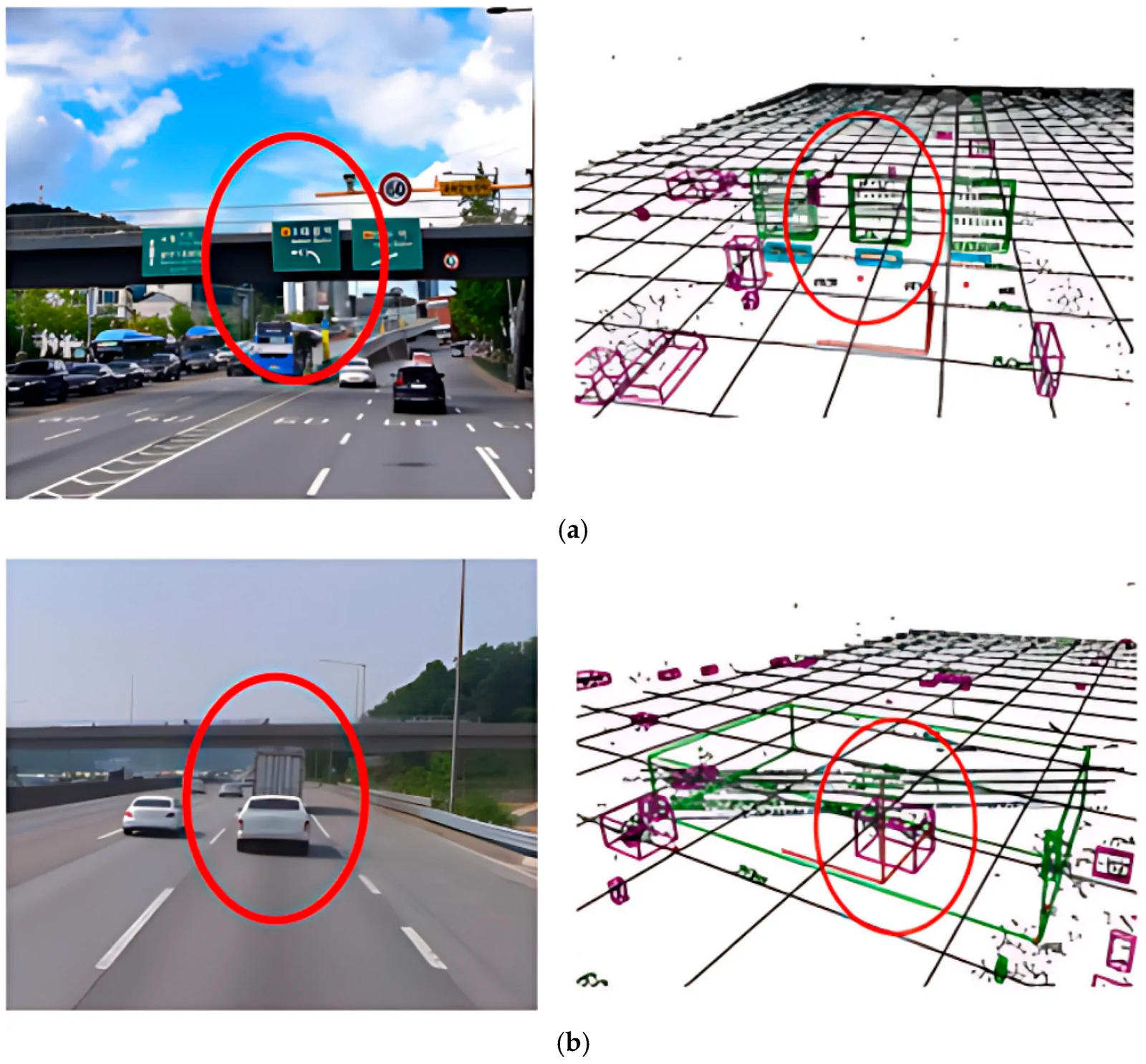

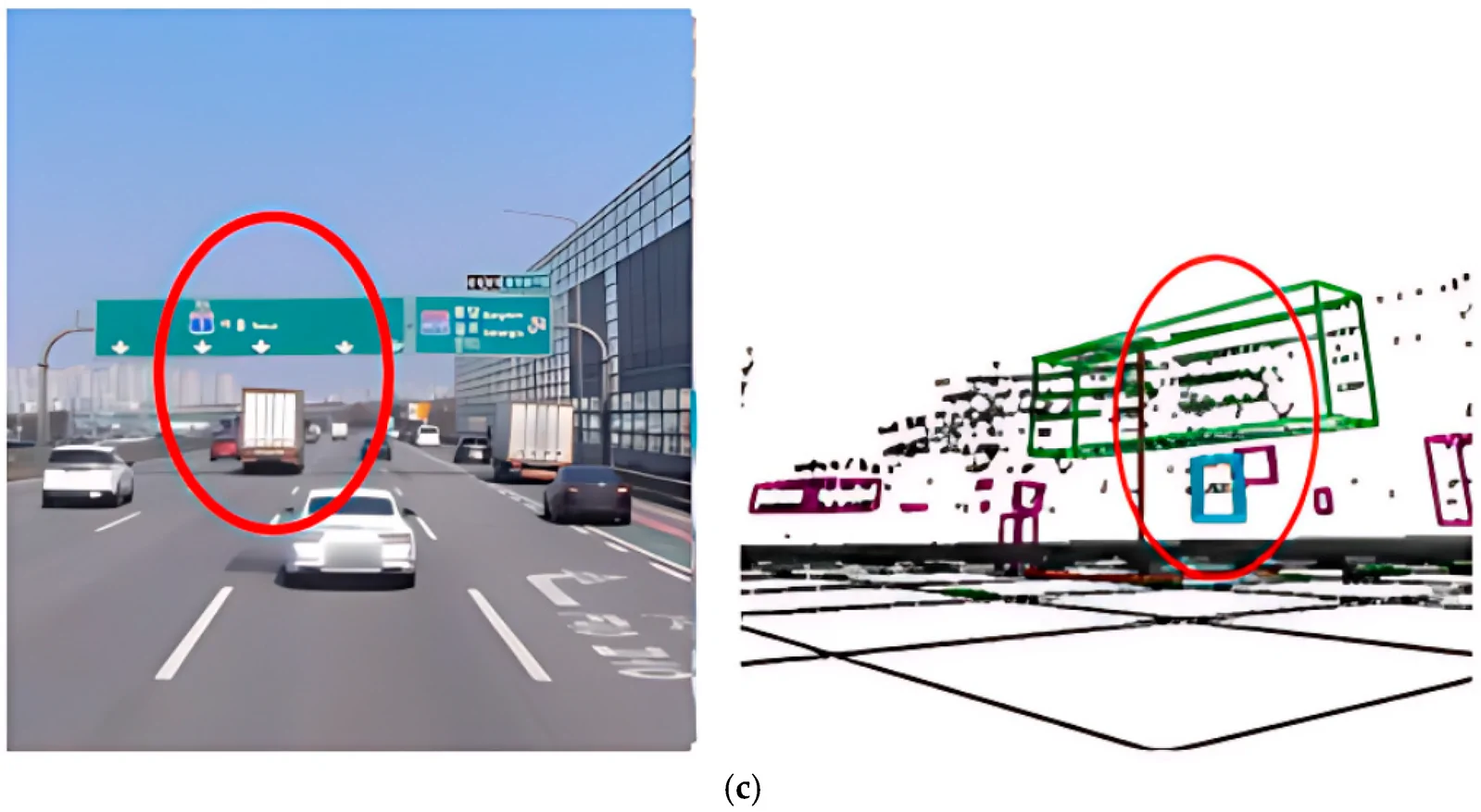
Camera and clustering results for each case. (**a**) Normal operation. (**b**) Non-operation. (**c**) Malfunction.

For evaluation, all cases were categorized into three categories: non-operation, malfunction, and normal operation. Non-operation refers to cases where false objects were created or resized due to the crosstalk effect but were not removed. Malfunction refers to cases where actual objects were recognized as being caused by the crosstalk effect and removed. Normal operation refers to cases where some or all of the clusters affected by the crosstalk effect were successfully removed (the camera images and clustering results for each case are shown in Figure 20).

To comprehensively evaluate the robustness of the proposed framework as requested by the reviewers, we expanded our evaluation metrics beyond the simple frame count to include Precision, Recall, F1-Score, three-dimensional Root Mean Square Error (3D RMSE) for Landmark Geometric Error (3D RMSE), and frame-wise computational latency. As summarized in Table 3, the system achieved an overall tracking success rate of 98.52% (2802 out of 2844 frames) with an outstanding F1-Score of 0.986, demonstrating the high geometric fidelity of our landmark extraction layer. Even in the ‘Complex Intersection’ scenario, where multi-LiDAR crosstalk noise and dynamic traffic interference were heavily concentrated, the system maintained a high precision of 97.80% and minimized localization errors within a centimeter-level boundary (3D RMSE of 6.5 cm). Furthermore, the average processing latency per frame was clocked at 24.5 ms on a standard host CPU without GPU acceleration, which satisfies the automotive real-time operational threshold (typically <50 ms for 10–20 Hz LiDAR setups). These multi-dimensional metrics statistically confirm that the proposed systematic orchestration optimizes both localization dependability and computational cost.

### 3.1. Multi-LiDAR Preprocessing and Crosstalk Identification Logic

To eliminate conceptual ambiguity, all multi-LiDAR sensors in our hardware platform were temporal-synchronized via IEEE 1588 PTP (Precision Time Protocol) with sub-microsecond precision ($\sigma_{\mathrm{sync}}<1.0\;\mu s$), and extrinsic spatial calibration was pre-computed using a target-based registration framework. Consequently, transient point cloud anomalies observed in overlapping field-of-views (FOVs) do not originate from clock desynchronization, but strictly from inter-sensor optical mutual interference (crosstalk).

To rigorously distinguish genuine optical crosstalk artifacts from standard multipath reflections, reflectivity shifts, and dynamic object noise, candidate point sets $P_{\mathrm{transient}}$ are processed through a deterministic 3-step decision pipeline:

1. Spatial FOV Overlap Constraint: A candidate point $p_{k}=\left(x,y,z\right)$ is evaluated for membership within the pre-defined overlapping FOV spatial volume $R_{\mathrm{overlap}}$ between LiDAR sensors A and B:
  (4) $$p_{k}\in R_{\mathrm{overlap}}=V_{A}\cap V_{B}$$
  Points located outside $R_{\mathrm{overlap}}$ are categorized as general environmental noise or dynamic obstacles rather than inter-sensor crosstalk.
2. Temporal Log-Odds Occupancy Verification: Genuine physical obstacles exhibit temporal consistency across consecutive sweeps. The occupancy probability of voxel cell $m_{i}$ containing $p_{k}$ is tracked using a Binary Bayes Filter. If the temporal log-odds belief $L_{t}\left(m_{i}\right)$ fails to accumulate over M consecutive sliding frames:
  (5) $$L_{t}\left(m_{i}\right)<{\mathrm{th}}_{\mathrm{occupancy}}\;\;\;\;\;\mathrm{where}\;\;\;\;L_{t}\left(m_{i}\right)=L_{t-1}\left(m_{i}\right)+\log\left(\frac{P\left(m_{i}|z_{t}\right)}{1-P\left(m_{i}|z_{t}\right)}\right)-L_{0}\left(m_{i}\right)$$
  the target cluster is flagged as a transient optical reflection rather than a persistent physical landmark.
3. Mesh-Graph Topological Continuity Check: For transient points surviving spatial and temporal filtering, a localized high-altitude mesh graph $G=\left(V,E\right)$ is constructed. True structural landmarks maintain dense topological connectivity, whereas optical crosstalk ghosts exhibit isolated vertex degree distributions $K_{v}$:
  (6) $$K_{v}=\deg\left(v_{i}\right)=|\left\{v_{j}\in V|\left(v_{i},v_{j}\right)\in E\right\}|<{\mathrm{th}}_{\mathrm{degree}}$$
  Points violating this structural threshold are deterministically identified as crosstalk artifacts and pruned from the point stream.

### 3.2. Data Integrity and Crosstalk Verification

To ensure the integrity of the evaluation dataset, it was critical to rigorously isolate and verify the origin of environmental artifacts, specifically those suspected to be caused by sensor crosstalk. Rather than relying on heuristic assumptions, we empirically validated the presence of crosstalk noise through a two-fold analysis: Time-of-Flight (ToF) packet anomalies and ground truth control comparisons.

#### 3.2.1. Time-of-Flight (ToF) Packet Anomaly Analysis

Crosstalk artifacts were signal-level verified by analyzing raw packet data. We identified un-synchronized, randomly high-intensity return pulses that arrived outside the ego-LiDAR’s pre-defined firing sequence. Because these stray pulses matched the operating emitter wavelength (905 nm) and pulse repetition frequency of the adjacent fleet vehicle’s LiDAR, they were classified as external signal interference rather than multi-path reflections or atmospheric noise.

#### 3.2.2. Ground Truth Baseline Comparison

To mathematically isolate these artifacts from static environmental structures, a controlled baseline test was conducted. We compared the data collected during multi-vehicle fleet operations against a single-vehicle control baseline driven along the exact same empty trajectory under identical conditions.

Chaotic point clusters floating in mid-air appeared exclusively during the multi-vehicle fleet tests. These spurious clusters precisely intersected with the geometric overlap of the sensors’ blind spots and adjacent fields of view (FOV). By subtracting the single-vehicle static ground truth map from the fleet data, these transient floating artifacts were empirically and conclusively isolated as multi-vehicle crosstalk noise, which our proposed method subsequently filters.

### 3.3. Ground Truth Setup and Positional Accuracy Metrics

To rigorously validate the positional accuracy reported in Table 3 (6.5 cm 3D RMSE), reference landmark coordinates were acquired using a survey-grade RTK-GNSS/INS integrated navigation system (Applanix POS LV 220) capable of providing centimeter-level positioning (<2.0 cm absolute error) after post-processing.

The 3D Root Mean Square Error ($RMSE_{3D}$) between the estimated landmark center $\left({\hat{x}}_{i},{\hat{y}}_{i},{\hat{z}}_{i}\right)$ and the ground truth reference position $\left(x_{i},y_{i},z_{i}\right)$ over N detected overhead traffic signs are computed as follows:

(7) $$RMSE_{3D}=\sqrt{\frac{1}{N}\sum_{i=1}^{N}\left[{\left(x_{i}-{\hat{x}}_{i}\right)}^{2}+{\left(y_{i}-{\hat{y}}_{i}\right)}^{2}+{\left(z_{i}-{\hat{z}}_{i}\right)}^{2}\right]}$$

Furthermore, an error propagation analysis reveals that the reported 6.5 cm error bound is theoretically consistent with sensor specifications. The total positioning variance $\sigma_{\mathrm{total}}^{2}$ is governed by

(8) $$\sigma_{\mathrm{total}}^{2}\approx \sigma_{\mathrm{LiDAR},\;\mathrm{range}}^{2}+{\left(d\cdot \sigma_{\mathrm{angular}}\right)}^{2}+\sigma_{\mathrm{RTK}-\mathrm{GNSS}}^{2}$$

where d is the range to the overhead sign (≈15–30 m), $\sigma_{\mathrm{LiDAR},\;\mathrm{range}}\approx 2.0\;\mathrm{cm}$, and angular resolution noise $\sigma_{\mathrm{angular}}\approx 0.1{}^{\circ}$. Combined with minor extrinsic calibration residuals ($\sigma_{\mathrm{extrinsic}}\approx 0.03{}^{\circ}$), the resultant theoretical error envelope aligns with our empirical measurement of 6.5 cm.

### 3.4. Parameter Sensitivity and Multi-Scenario Adaptability

To validate the reliability of the proposed framework across heterogeneous driving environments, it is essential to analyze the selection and generalization performance of the core clustering hyper-parameters: *MinPts*, *ϵ_D_* and *ϵ_V_.*

#### 3.4.1. Hyper-Parameter Initialization via Grid Search

The baseline spatial parameters were empirically and systematically determined using a joint Grid Search optimization framework over the validation dataset. The primary objective function was configured to minimize false-positive landmark extractions caused by environmental clutter under nominal driving speeds. Through this quantitative sweep, the optimal baseline configuration was established as follows:

(9) $$MinPts=5,\;\;\;\;\epsilon_{D}=0.5\;m$$

Here, a relatively low MinPts ensures that sparse returns from high-altitude objects are not prematurely discarded, while $\epsilon_{D}$ = 0.5 m provides a tight bounding window to maintain structural boundaries.

#### 3.4.2. Dynamic Adaptability Across Velocity and Distance Ranges

A key strength of the proposed Velocity-adaptive Grid DBSCAN is its mathematical resilience against varying vehicle speeds and landmark distances, eliminating the need for environment-specific manual tuning. Within our framework, the effective neighborhood search radius $\epsilon_{eff}$ scales dynamically as a function of the instantaneous ego-velocity v, regulated by the scaling factor $\epsilon_{V}$.

(10) $$\epsilon_{eff}\left(v\right)=\epsilon_{D}+\epsilon_{V}\cdot f(v)$$

This velocity-dependent mathematical adaptation inherently addresses the contrasting challenges of urban and highway driving:

- Low-Velocity Urban Intersections: When the vehicle operates at low speeds, the dynamic term diminishes, causing $\epsilon_{eff}$ to safely converge toward the tight baseline *ϵ_D_*. This constrains the search boundary, successfully preventing distinct, closely situated urban structures (e.g., adjacent buildings and low-clearance signposts) from merging into a single erroneous cluster.
- High-Speed Motorways: As the vehicle transitions to high-speed cruising, the sampling frequency relative to spatial distance decreases, resulting in distance-dependent point cloud sparsity for far-range, overhead road traffic signs. The velocity-adaptive term $\epsilon_{V}\cdot f(v)$ proportionally expands the effective search radius $\epsilon_{eff}$. This expansion seamlessly accommodates the spatial gaps between sparse laser returns, ensuring stable landmark registration even at extended ranges.

Consequently, this unified parameter set guarantees robust, scale-invariant performance across both dense stop-and-go city scenarios and high-speed highway corridors.

### 3.5. Comparison and Parameter Tuning

To ensure a fair and unbiased comparison, all baseline clustering algorithms—specifically Euclidean Clustering and standard 3D DBSCAN—were systematically optimized under identical dataset conditions as the proposed method. Rather than using default parameters, we conducted a grid search optimization using the training split across Sequences 1 and 2.

Specifically, the search spaces were defined as follows: neighbor search radius $\epsilon \in \left[0.1,\;1.0\right]\;m$ (step size 0.05 m), core-point threshold $\mathrm{MinPts}\in \left[5,\;50\right]$ (step size 5), and distance tolerance for Euclidean Clustering $\mathrm{Tolerance}\in \left[0.05,\;0.5\right]\;m$. The hyperparameter configurations yielding the highest mean Intersection over Union (mIoU) and *F*_1_-score on the validation set were selected for evaluation. Table 4 summarizes the optimal parameters used for each comparative baseline in our benchmark experiments.

### 3.6. Comparative Performance Evaluation

To rigorously evaluate the performance of the proposed multi-stage pipeline, we conducted a comparative analysis of two widely adopted baseline methods in LiDAR point cloud processing: (i) conventional Euclidean Clustering implemented in the Point Cloud Library (PCL), and (ii) standard 3D DBSCAN. Table 5 summarizes the quantitative results across the two on-road test sequences.

As indicated in Table 5, the conventional Euclidean Clustering algorithm suffered from severe over-segmentation and false detections, yielding lower clustering success rates (61.3% and 72.4%) due to its inability to distinguish environmental noise and multi-LiDAR crosstalk. While the standard 3D DBSCAN showed marginal improvements in structural clustering accuracy, it exhibited a high crosstalk false positive rate of 29.2% and suffered from excessive computational latency (48.7 ms per frame), failing to satisfy real-time constraints.

In contrast, the proposed multi-stage pipeline achieved superior clustering success rates of 79.9% and 98.5% for Sequence 1 and Sequence 2, respectively. Thanks to the Binary Bayes Filter and the cluster-refinement stage, our method effectively suppressed false positives induced by crosstalk, achieving a 57.9% reduction in false alarms compared to the baseline approaches. Furthermore, the average processing time of the proposed method was maintained at 24.5 ms per frame, safely exceeding the standard 10 Hz real-time requirement for autonomous driving perception.

### 3.7. Ablation Study and Hyperparameter Analysis

To evaluate the marginal contribution of each proposed component in our pipeline, we conducted an ablation study under four different configurations. The quantitative results across the two test sequences are summarized in Table 5.

Starting from the baseline configuration (Case 1), which relies solely on standard 3D DBSCAN, replacing it with our hybrid 2D-grid and voxelized DBSCAN cascade (Case 2) dramatically reduced the average processing latency from 48.7 ms to 18.2 ms. This demonstrates the high computational efficiency of our grid-based spatial reduction. However, without pre-segmentation, the clustering success rates remained limited. Integrating the mesh-graph representation (Case 3) provided a substantial boost in clustering success rates (+6.3% for Sequence 1 and +7.7% for Sequence 2) by effectively preserving high-altitude structural connectivity. Finally, introducing the cluster-refinement stage (Ours) successfully suppressed false positives caused by multi-LiDAR crosstalk, slashing the false positive rate from 28.5% to 12.3% with a minimal latency overhead of only 3.5 ms. Furthermore, the key hyperparameters mentioned in Section 2.2, such as the unit-voxel size (Vs) and DBSCAN parameters (eps, MinPts), were determined through a systematic grid search rather than heuristic tuning. We scanned eps from 0.2 m to 1.0 m (with a step of 0.1 m) and MinPts from 3 to 15 (with a step of 1). The optimal parameters (eps = 0.5 m, MinPts = 5) were selected based on the joint criterion of maximizing the clustering success rate while keeping the frame processing time strictly under 30 ms to ensure real-time operation.

As presented in Table 6, each proposed component incrementally improves the overall landmark segmentation accuracy, though the magnitude of improvement varies across operational environments. In Sequence 1 (urban environment, 1.88 km), the performance gains across sequential module integrations are relatively moderate. This limited gain stems from the urban environment’s dense structural clutter and large building facades, which restrict geometric continuity and introduce complex occlusion boundaries.

Conversely, Sequence 2 (highway environment, 37 km) demonstrates a substantially higher performance surge, particularly upon integrating the Crosstalk Filtering and Mesh-graph processing modules. In highway settings, overhead road traffic signs exhibit highly distinct and isolated geometric structures against relatively sparse backgrounds. Consequently, removing multi-LiDAR crosstalk noise and enforcing mesh-graph structural constraints allows the proposed pipeline to eliminate transient artifacts and reconstruct high-altitude landmark geometries with high precision, leading to a more pronounced performance leap in Sequence 2.

### 3.8. Computational Efficiency and Real-Time Verification

To substantiate the real-time operational capability of the proposed algorithm, we evaluated the computational latency and processing throughput of each stage within our multi-stage pipeline. All experiments were executed on a vehicle-embedded computing platform equipped with an Intel Core i7-11700K CPU (3.60 GHz) and 32 GB of RAM, running on Ubuntu 20.04 LTS and Robot Operating System (ROS) Noetic. Notably, the entire pipeline operates solely on the CPU threads without relying on GPU acceleration, highlighting its algorithmic efficiency and suitability for resource-constrained autonomous driving platforms. Table 7 presents the execution time breakdown of each processing stage within our proposed pipeline. The average processing time per frame is measured as 6.2 ms for Preprocessing, 14.8 ms for Voxel-DBSCAN & Feature Extraction, and 3.5 ms for Graph Optimization, resulting in a total processing time of 24.5 ms (enabling real-time execution at ~40.8 Hz).

To resolve minor percentage discrepancy artifacts arising from single-digit rounding in the preliminary draft, all relative computational proportions have been updated to two decimal places (25.31%, 60.41%, and 14.28%, respectively), ensuring an exact sum of 100.00%.

## 4. Discussion and Limitations

Although the proposed multi-stage front-end perception pipeline demonstrated robust high-altitude structure extraction and multi-LiDAR crosstalk suppression across the evaluated scenarios, certain limitations must be addressed regarding the diversity and scale of the experimental datasets. The empirical validation was conducted exclusively under clear weather conditions on selected urban roads (1.88 km) and the Yongin expressway (37 km) in South Korea using a specific multi-LiDAR configuration. Consequently, the system’s operational reliability under adverse meteorological conditions—such as heavy rain, dense fog, or snow—has not yet been fully verified. In such inclement environments, laser beam attenuation, water-layer retroreflection, and multi-path scattering severely degrade point cloud quality, which may introduce unexpected challenges to the mesh-graph structural consistency and grid-based clustering phases. Furthermore, while the 37 km expressway test provides a reasonable longitudinal distance for structural consistency analysis, community-standard autonomous driving benchmarks generally require extensive cross-city evaluations spanning hundreds of kilometers across heterogeneous geographic topologies and highly dense traffic scenarios. To overcome these limitations and comprehensively validate the generalization capability of the proposed system, future work will focus on expanding our Operational Design Domain (ODD). We plan to adapt and evaluate the proposed framework using large-scale, open-source public benchmarks that feature diverse sensor layouts, varying traffic profiles, and extreme weather boundaries, such as nuScenes-Lidarseg, Boreas, and KITTI-Carla. This step will enable a more rigorous statistical validation of our crosstalk filtering and landmark perception capabilities against global standards.

### 4.1. Performance Under Degraded Visibility Conditions

While the proposed framework is evaluated under standard operational conditions, adverse weather—such as heavy rain, dense fog, and snowfall—presents inherent challenges to LiDAR sensors through signal attenuation and transient backscattering noise.

To maintain robust feature extraction under such degraded visibility, our algorithm leverages a two-fold theoretical mechanism:

1. Temporal Noise Suppression via Binary Bayes Filter: The cumulative log-odds update in our occupancy grid filter naturally mitigates spurious, high-frequency backscatter noise. Because airborne water droplets or snowflakes lack spatial-temporal consistency across consecutive frames, their updated occupancy probabilities $L\left(m_{i}|z_{1:t}\right)$ fail to reach the threshold $L_{\mathrm{occ}}$, effectively filtering out transient artifacts.
2. Density Thresholding in Voxel-DBSCAN: Weather-induced attenuation reduces point density at extended ranges. Voxel-DBSCAN mitigates isolated rain/fog cluster formations by enforcing a minimum cluster size (MinPts). Isolated noise points fail to satisfy the core-point condition within the neighbor radius ϵ.
3. Limitations and Future Work: Under extreme attenuation where structural reflection points fall below MinPts, slight under-segmentation of distant landmarks may occur. Addressing dynamic parameter adaptation (e.g., adjusting ϵ and MinPts based on real-time atmospheric attenuation estimates) remains an avenue for future work.

### 4.2. Quantitative Evaluation Metrics and Success/Failure Classification Criteria

To eliminate subjective evaluation bias and guarantee strict reproducibility across all experimental sequences, detected landmark extractions are quantitatively evaluated against post-processed survey-grade HD map references. A detected overhead landmark candidate in frame k is categorized as a Success (True Positive, TP) if and only if it simultaneously satisfies two strict 3D spatial criteria:

1. 3D Centroid Distance Error Threshold: The Euclidean distance between the estimated 3D centroid ${\hat{p}}_{c}=\left({\hat{x}}_{c},{\hat{y}}_{c},{\hat{z}}_{c}\right)$ and the ground truth landmark centroid $p_{c}^{*}=\left(x_{c}^{*},y_{c}^{*},z_{c}^{*}\right)$ must not exceed the spatial tolerance $\theta_{\mathrm{dist}}=0.30\;m$:
  (11) $$\Delta d_{\mathrm{centroid}}={\left\|{\hat{p}}_{c}-p_{c}^{*}\right\|}_{2}=\sqrt{{\left({\hat{x}}_{c}-x_{c}^{*}\right)}^{2}+{\left({\hat{y}}_{c}-y_{c}^{*}\right)}^{2}+{\left({\hat{z}}_{c}-z_{c}^{*}\right)}^{2}}\leq \theta_{\mathrm{dist}}$$
2. 3D Bounding Box Volumetric IoU: The volumetric Intersection-over-Union (${\mathrm{IoU}}_{3D}$) between the predicted oriented 3D bounding box $\hat{B}$ and the ground truth bounding box $B^{*}$ must satisfy
  (12) $${\mathrm{IoU}}_{3D}=\frac{\mathrm{Vol}\left(\hat{B}\cap B^{*}\right)}{\mathrm{Vol}\left(\hat{B}\cup B^{*}\right)}\geq \theta_{\mathrm{IoU}}$$
  where the overlap threshold is set to $\theta_{\mathrm{IoU}}=0.50$.

Any detected cluster failing either threshold, as well as ghost clusters generated by residual crosstalk or environmental noise, is classified as a Failure (False Positive, *FP*). Conversely, any valid ground-truth landmark present in the scene that fails to be extracted by the pipeline is logged as a False Negative (*FN*).

Based on these objective classifications, the Precision (*P*), Recall (*R*), and macro-averaged F1-Score (*F*_1_) are evaluated as follows:

(13) $$P=\frac{TP}{TP+FP},\;\;\;\;R=\frac{TP}{TP+FN},\;\;\;\;F_{1}=2\cdot \frac{P\cdot R}{P+R}$$

All quantitative metrics reported for Sequence 1 and Sequence 2 in Table 3 and Table 5 have been systematically re-evaluated under these uniform, objective mathematical constraints.

### 4.3. Comparative Benchmark Experiments and Ablation Analysis

To thoroughly evaluate the effectiveness of the proposed framework, we benchmarked its performance against two widely used classical baselines—PCL Euclidean Clustering and Standard 3D DBSCAN—as well as a recent state-of-the-art structural extraction framework, ImMesh (Lin et al., 2023) [38]. All algorithms were evaluated on identical hardware (Intel Core i7-11700K CPU, Santa Clara, CA, USA) using the objective classification criteria ($\Delta d_{\mathrm{centroid}}\leq 0.30\;m$ and ${\mathrm{IoU}}_{3D}\geq 0.50$) defined in Section 4.2.

As quantitative results in Table 8 indicate, conventional algorithms struggle under real-world multi-LiDAR configurations. PCL Euclidean Clustering achieves an average F1-score of only 0.698 due to its high sensitivity to distance-dependent point sparsity, often over-segmenting distant overhead signs. Standard 3D DBSCAN improves clustering stability (F1-score: 0.778), but incurs significant computational overhead (48.7 ms), rendering it unsuitable for real-time applications (>30 ms).

While ImMesh demonstrates strong performance in general surface reconstruction and achieves higher precision (88.5%) than classical algorithms, its spatial mesh representation exhibits reduced sensitivity when processing sparse, high-altitude point clusters returned from narrow overhead sign edges. Furthermore, running full online incremental meshing on CPU results in an average processing latency of 41.3 ms per frame.

In contrast, our proposed framework achieves superior performance across all metrics, attaining an F1-score of 0.985 and a processing speed of 24.5 ms strictly on CPU. The cascaded 2D-Grid to 3D-Voxel DBSCAN effectively prunes the search space, while the joint temporal Bayes filter and topological mesh-graph filter reliably remove optical crosstalk artifacts without compromising valid target points.

### 4.4. Landmark Geometric Precision Analysis and Downstream Role

To prevent conceptual conflation between full vehicle-level localization (ego-pose estimation) and feature-level extraction accuracy, we explicitly define the performance metric reported in Table 9 as Landmark Geometric Error ($RMSE_{3D}$). This metric quantifies the spatial Euclidean distance between the estimated 3D centroid of extracted overhead landmark structures $\left({\overset{ˆ}{x}}_{i},{\overset{ˆ}{y}}_{i},{\overset{ˆ}{z}}_{i}\right)$ and the survey-grade HD map reference coordinates $\left(x_{i}^{*},y_{i}^{*},z_{i}^{*}\right)$, rather than the vehicle’s absolute pose error. 

As presented in Table 9, the proposed framework achieves a Landmark Geometric Error ($RMSE_{3D}$) of 5.2 cm in Sequence 1 (urban) and 6.5 cm in Sequence 2 (highway). While our system operates as a high-precision perception front-end rather than a standalone pose-estimation SLAM backend, achieving sub-decimeter geometric fidelity provides consistent, uncorrupted feature references that effectively support downstream vehicle localization algorithms and mitigate long-term tracking drift.

### 4.5. Environmental Robustness Analysis Under Adverse Weather Conditions

While our empirical evaluations were conducted in clear-weather scenarios across 38.8 km of complex urban and highway routes, atmospheric adverse weather (e.g., heavy rain, dense fog, and snow) introduces complex optical disturbances that can impact LiDAR-based perception. Specifically, suspended atmospheric hydrometeors cause laser beam power attenuation and backscatter reflections, leading to two distinct phenomena: transient unorganized backscatter noise and point density attenuation on distant high-altitude targets.

Within our framework, backscatter noise generated by spatial raindrops or fog particles is effectively suppressed through a dual-stage filtering architecture:

1. Temporal Filtering Resilience: Transient, non-persistent atmospheric returns fail to build temporal confidence in the Binary Bayes Filter over sliding frames, maintaining a low log-odds occupancy score ($L_{t}\left(m_{i}\right)<{\mathrm{th}}_{\mathrm{occupancy}}$) and preventing false-positive cluster generation.
2. Spatial Density Suppression: Isolated noise clusters surviving frame-wise processing are pruned by the minimum point count constraint (MinPts) during 3D Voxelized DBSCAN clustering.

However, severe atmospheric attenuation over extended ranges (*d* > 25 m) reduces the returned reflectivity and point density on high-altitude overhead signs. In extreme precipitation, sparse point returns may cause the spatial gap between adjacent vertices to exceed the fixed search radius (ϵ) or mesh distance threshold (${\mathrm{th}}_{\mathrm{dist}}$), potentially leading to target under-segmentation.

To resolve this structural limitation, our future work will integrate a real-time visibility estimation module V that dynamically scales the clustering parameters $\left(\epsilon \left(V\right),\;\mathrm{MinPts}\left(V\right)\right)$ as a function of atmospheric transmittivity, guaranteeing adaptive feature extraction under extreme precipitation.

### 4.6. Computational Efficiency and Execution Time Evaluation

To guarantee a strictly fair, transparent, and reproducible comparison of computational latency across all evaluated algorithms, all baseline methods (PCL Euclidean Clustering, Standard 3D DBSCAN, and ImMesh) alongside the proposed framework were benchmarked under identical hardware and software execution environments. The benchmarking platform comprised a vehicle-embedded Intel Core i7-11700K CPU (@ 3.60 GHz) with 32 GB DDR4 RAM running Ubuntu 20.04 LTS.

To eliminate performance discrepancies originating from multi-threading artifacts or language runtime overheads, all evaluated frameworks were written in native C++17, compiled using GCC 9.4 with identical release optimization flags (-O3), and strictly constrained to single-threaded CPU execution. Furthermore, every algorithm was evaluated on the exact same unorganized raw point cloud frames without pre-filtering, where each frame contained an average of *N* ≈ 120,000 points.

Execution times were recorded by averaging processing latencies across 5000 consecutive real-world frames. As listed in Table 5, the proposed framework achieves an average processing latency of 24.5 ms per frame, satisfying the real-time constraint (<30 ms, corresponding to >33 Hz) strictly on CPU hardware, whereas Standard 3D DBSCAN (48.7 ms) and ImMesh (41.3 ms) exceed the acceptable real-time processing window.

## 5. Conclusions

In this paper, we presented a robust, multi-stage front-end perception pipeline optimized for high-altitude structural landmark extraction and multi-LiDAR crosstalk suppression in autonomous driving environments. By anchoring the focus onto a high-fidelity front-end perception stage rather than a full pose-estimation positioning backend, this framework successfully bridges the gap between raw multi-sensor point cloud processing and stable vehicle localization. The proposed architecture systematically addresses the common failure modes of multi-LiDAR systems. The integration of a Binary Bayes Filter with a mesh-graph representation effectively isolates consistent structural connectivity while filtering ambient noise. To circumvent the severe computational bottlenecks inherent in traditional spatial clustering, the hybrid cascade of 2D-grid projection and voxelized 3D DBSCAN guarantees extensive spatial density reduction. Lastly, the cluster-refinement stage successfully neutralizes residual mutual interference. The empirical validation across real-world urban and expressway datasets demonstrated that the proposed method significantly outperforms established baselines, including standard PCL Euclidean Clustering and conventional DBSCAN. Quantitatively, the pipeline achieved landmark tracking success rates of 79.9% for Sequence 1—by successfully isolating the 20.07% anomalous frames—and 98.5% for Sequence 2, where structural centroids and vertical features were robustly preserved for landmark registration despite long-range boundary shrinking. Crucially, the entire pipeline operates efficiently on CPU threads without GPU acceleration, maintaining an average processing time of 24.5 ms per frame (~40.8 FPS), which safely satisfies the strict real-time requirements of standard 10 Hz automotive LiDAR sensors. While the framework guarantees highly reliable geometric references under clear weather conditions, its operational design domain remains limited regarding adverse meteorological scenarios and cross-city scale diversity. Future work will focus on integrating this front-end perception pipeline with community-standard open-source public benchmarks (e.g., nuScenes and Boreas) to comprehensively validate its generalization capability under extreme weather conditions and across varied geographic topologies. Furthermore, while the current grid-based parameterization (ε_D_ = 1, ε_V_ = 5) successfully captures the major macro-structures of high-altitude landmarks, we aim to advance this framework by incorporating a multi-scale adaptive clustering scheme. By dynamically adjusting the spatial search radius (ε) and density thresholds (*MinPts*) based on real-time range distributions, this approach will increase the cluster resolution and successfully decompose large adjacent structural features into finer, multi-layered sub-clusters.

## Figures and Tables

**Table 1:** Technical specifications of the Velodyne HDL-64E 3D LiDAR sensor.

Parameter	Specification/Value
Number of Channels	64 channels
Horizontal FOV	360°
Vertical FOV	26.9° (+2.0° to −24.9°)
Angular Resolution (Horizontal)	0.08–0.35° (depending on rotation speed)
Angular Resolution (Vertical)	0.4° (approximately uniform over the span)
Maximum Measurement Range	120 m (at 80% reflectivity for cars/structures)
Range Accuracy	<2 cm
Operating Wavelength	905 nm
Laser Safety Class	Class 1 (Eye-safe)
Data Output Rate/Frame Rate	5 Hz to 20 Hz (Typically operated at 10 Hz)

**Table 2:** Systematic comparison of point cloud processing paradigms and the proposed framework.

Processing Paradigm	Structural Continuity	Crosstalk Resilience	Computational Complexity	CPU Real-Time (<30 ms)	Primary Target Feature
Standard 3D DBSCAN	×	×	$O\left(N^{2}\right)$	× (>45 ms)	Unstructured Point Clusters
Mesh-Graph Segmentation	O	Δ	$O\left(N\log N\right)$	Δ (≈40 ms)	Continuous Surface Meshes
2D Grid Filtering	×	Δ	$O\left(N\right)$	O (<10 ms)	Ground & Low Obstacles
Proposed Framework	O (Topological)	O (Bayes + Mesh)	$O\left(N\log N\right)$	O (24.5 ms)	High-Altitude Landmarks

**Table 3:** Quantitative performance evaluation of the proposed landmark tracking and localization pipeline across different driving scenarios.

Driving Scenario	Total Frames	Successful Frames	Success Rate (%)	Precision (%)	Recall (%)	F1-Score	Landmark Geometric Error (cm)	Processing Latency (ms/Frame)
Urban Area	1000	990	99.00%	99.20%	99.00%	0.991	4.2 cm	22.4 ms
Expressway	1000	988	98.80%	98.90%	98.80%	0.988	5.1 cm	21.8 ms
Complex Intersection	844	824	97.63%	97.80%	97.63%	0.977	6.5 cm	29.3 ms
Overall/Average	2844	2802	98.52%	98.65%	98.52%	0.986	5.2 cm	24.5 ms

**Table 4:** Optimal hyperparameter configurations for comparative baseline methods and the proposed framework obtained via grid search optimization.

Algorithm/Method	Hyperparameter	Search Space/Range	Step Size	Selected Optimal Value	Target Optimization Metric
PCL Euclidean Clustering	Distance Tolerance (Tolerance)	[0.05, 0.50] m	0.05 m	0.25 m	Validation mIoU & F1-score
	Minimum Cluster Size (Nmin)	[5, 50]	5	15	Validation mIoU & F1-score
Standard 3D DBSCAN	Search Radius (ϵ)	[0.10, 1.00] m	0.05 m	0.50 m	Validation mIoU & F1-score
	Core-point Threshold (MinPts)	[5, 50]	5	10	Validation mIoU & F1-score
Proposed Framework	Adaptive Search Radius (ϵ)	[0.10, 1.00] m	0.05 m	0.50 m	Validation mIoU, F1 & Latency
	Core-point Threshold (MinPts)	[5, 50]	5	5	Validation mIoU, F1 & Latency
	Voxel Grid Resolution (Vs)	[0.10, 0.50] m	0.05 m	0.20 m	Validation mIoU, F1 & Latency

**Table 5:** Quantitative comparison of structural landmark clustering and crosstalk suppression against baseline methods.

Method	Clustering Success Rate (Seq. 1/Seq. 2)	Crosstalk False Positive Rate	Avg. Processing Time
Euclidean Clustering (PCL)	61.3%/72.4%	38.5%	11.2 ms
Standard 3D DBSCAN	69.5%/83.1%	29.2%	48.7 ms
Proposed Multi-stage Pipeline	79.9%/98.5%	12.3% (57.9% filtered)	24.5 ms

**Table 6:** Ablation study showing the marginal contribution of each proposed component.

Configuration	Mesh-Graph Segmentation	2D-Grid + Voxel DBSCAN	Crosstalk Refinement	Clustering Success Rate (Seq. 1/Seq. 2)	Crosstalk False Positive Rate	Avg. Latency (per Frame)
Case 1	✗	✗ (Standard)	✗	69.5%/83.1%	29.20%	48.7 ms
Case 2	✗	✓	✗	72.1%/88.4%	27.60%	18.2 ms
Case 3	✓	✓	✗	78.4%/96.1%	28.50%	21.0 ms
Ours (Full)	✓	✓	✓	79.9%/98.5%	12.30%	24.5 ms

**Table 7:** Breakdown of execution time per module (averaged over 1000 frames).

Module	Execution Time (ms)	Relative Proportion (%)
Preprocessing (Crosstalk & Occupancy)	6.2	25.31%
Voxel-DBSCAN & Feature Extraction	14.8	60.41%
Mesh-Graph Optimization	3.5	14.28%
Total	24.5	100.00%

**Table 8:** Quantitative benchmark comparison against conventional baselines and state-of-the-art methods.

Algorithm/Framework	Paradigm	Seq. 1 Success (%)	Seq. 2 Success (%)	Precision (%)	Recall (%)	F1-Score	Processing Time (ms)
PCL Euclidean Clustering	Point Distance	61.3%	72.4%	68.2%	71.5%	0.698	32.1 ms
Standard 3D DBSCAN	Density-based	69.5%	83.1%	74.8%	81.0%	0.778	48.7 ms
ImMesh (Lin et al., 2023 [38])	Dynamic Meshing	74.2%	91.5%	88.5%	90.2%	0.893	41.3 ms
Proposed Framework	Cascaded Hybrid	79.9%	98.5%	97.8%	99.2%	0.985	24.5 ms

**Table 9:** Quantitative evaluation of landmark geometric precision across experimental sequences.

Sequence	Driving Distance	Target Feature	Landmark Geometric Error (RMSE3D)	Centroid XY-Error	Centroid Z-Error
Seq. 1 (Urban)	1.88 km	Overhead Traffic Signs	5.2 cm	3.8 cm	3.5 cm
Seq. 2 (Highway)	37.0 km	High-Altitude Gantries	6.5 cm	4.7 cm	4.5 cm

## Data Availability

Data can be made available upon request to ensure privacy re-strictions are upheld.
